# RUNX1-ETO oncoprotein levels determine the balance between endothelial reprogramming and pre-leukemic growth in a human ES cell model of AML

**DOI:** 10.1016/j.celrep.2026.118157

**Published:** 2026-10-19

**Authors:** Katerina Terolli, Floyd Hassenrueck, Alexander Maytum, Peter Keane, Lea Flippe, Sophie G. Kellaway, Merel van der Meulen, Olaf T. Heidenreich, Edouard G. Stanley, Elizabeth S. Ng, Andrew G. Elefanty, Constanze Bonifer

**Affiliations:** 1 Murdoch Children’s Research Institute, The Royal Children’s Hospital, Parkville, VIC 3052, Australia; 2 School of Biosciences, College of Life and Environmental Sciences, University of Birmingham, Birmingham B15 2TT, UK; 3 Department of Cancer and Genomic Sciences, School of Medical Sciences, College of Medicine and Health, University of Birmingham, Birmingham B15 2TT, UK; 4 Blood Cancer and Stem Cell Group, University of Nottingham, Nottingham NG7 2RD, UK; 5 The Novo Nordisk Foundation Center for Stem Cell Medicine (reNEW), Murdoch Children’s Research Institute, Parkville, VIC 3052, Australia; 6 Department of Paediatrics, Faculty of Medicine, Dentistry and Health Sciences, University of Melbourne, Parkville, VIC 3052, Australia; 7 Princess Maxima Centre of Pediatric Oncology, 3584 CS Utrecht, the Netherlands

**Keywords:** t(8;21) acute myeloid leukemia, RUNX1-ETO and RUNX1 interplay, Lineage inappropriate gene activation, single cell gene expression analysis, endothelial reprogramming, embryonic stem cell differentiation into hematopoietic stem cells, regulation of leukemia growth by endothelial cells

## Abstract

In t(8;21) acute myeloid leukemia (AML), the RUNX1 DNA binding domain is fused to RUNX1T1 (ETO) producing the RUNX1-ETO fusion protein. We previously showed that t(8;21) leukemic stem cells aberrantly activate endothelial signaling pathways to initiate blast cell proliferation. Here, we employed a human embryonic stem cell (ESC) line carrying an inducible RUNX1-ETO transgene to determine whether endothelial signaling pathway activation depends on disease progression or is directly induced by RUNX1-ETO. Single-cell analyses demonstrate that RUNX1-ETO reprograms ESC-derived myeloid progenitors towards endothelial cells. We show (1) that endothelial reprogramming is RUNX1-ETO concentration-dependent and irreversible, (2) at similar RUNX1-ETO and RUNX1 expression levels, a sub-population of blood progenitors can escape reprogramming, and (3) keeps proliferating when co-cultured with endothelial cells. Our experiments highlight the role of cellular interactions in the maintenance of pre-leukemic cells and provide important insights into the earliest stages of genomic reprogramming by oncogenic transcription factors.

## Highlights

• Elevated RUNX1-ETO expression turns human ESC-derived HSCs into endothelial cells
• Endothelial reprogramming is irreversible but incomplete
• Hematopoietic progenitors escape growth arrest at similar RUNX1-ETO/RUNX1 levels
• Endothelial cells expressing stem cell factor support growth of these cells

## Graphical abstract

**Figure:**
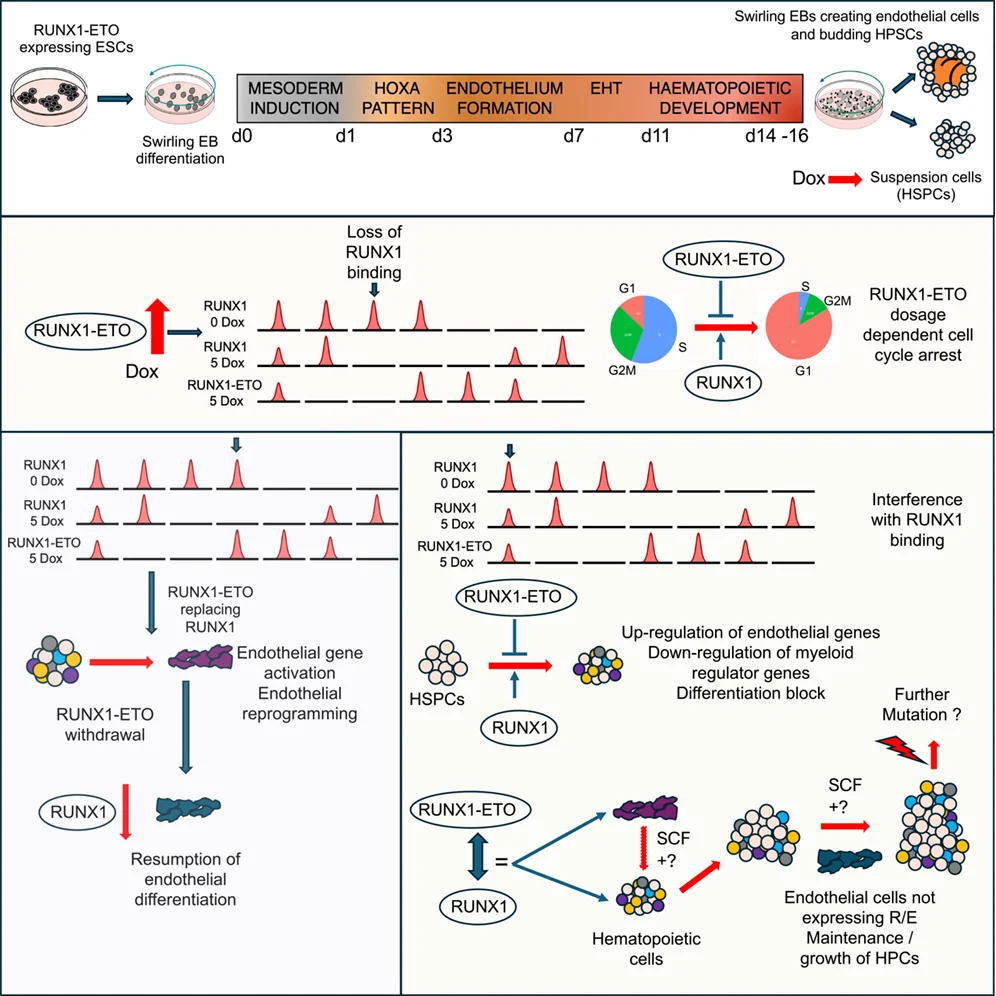

## Teaser

Terolli et al. investigated early leukemogenesis using human embryonic stem cells harboring an inducible RUNX1-ETO oncogene differentiated toward blood. High oncogene expression induced cell-cycle arrest and irreversible myeloid-to-endothelial reprogramming. Balanced expression with RUNX1 allowed a sub-population of hematopoietic progenitors to escape reprogramming and growth arrest when co-cultured with endothelial cells.

## Introduction

Acute myeloid leukemia (AML) is caused by multiple mutations which alter the function of transcription factors (TFs), chromatin modifiers, or signaling molecules, impacting on hematopoietic differentiation. AML driven by the t(8;21) chromosomal translocation is one of the best characterized subtypes.^1^ The translocation produces the RUNX1-ETO (R/E) oncoprotein by fusing the DNA binding domain of RUNX1 to the RUNX1T1 (ETO) protein. Transcription of the fusion gene is regulated by the *RUNX1* promoters and is therefore active at the same developmental stages as the wild-type *RUNX1* gene. Importantly, R/E interferes with the normal action of RUNX1 by binding to the same binding motifs in the genome.^2^^,^^3^^,^^4^ Indeed, similar to the phenotype observed in *Runx1* knock out mice, germline expression of R/E results in embryonic lethality with a complete lack of fetal liver hematopoiesis.^5^^,^^6^^,^^7^^,^^8^ Mice expressing full length R/E in hematopoietic cells show perturbed hematopoietic differentiation characterized by expansion of the hematopoietic stem and progenitor cell (HSPC) compartment and the eventual development of an indolent myeloproliferative disorder.^9^^,^^10^ This phenotype supports the conclusion that expression of R/E alone is not sufficient to cause AML, and that additional co-operating mutations are required for oncogenic transformation.^11^^,^^12^ Commonly, these secondary mutations affect genes encoding signaling molecules, exemplified by activating mutations in the stem cell factor (SCF) receptor, KIT.^13^

An important feature of the pathology of t(8;21) AML is that the translocation can be found from an early stage of embryonic development onwards.^14^ Several studies have demonstrated the importance of the developmental stage at which the translocation occurs. Hematopoietic stem cells (HSCs) and their immediate precursors arise from the dorsal aorta of the vertebrate embryo via a process called the endothelial-hematopoietic transition (EHT) whereby newly formed blood cells enter circulation and take up residence in the fetal liver. This temporary site of hematopoiesis is subsequently extinguished when cells migrate to the bone marrow, where they establish long-term adult hematopoiesis.^15^ The EHT involves (1) the up-regulation of *RUNX1* in hemogenic endothelial cells, (2) the activation of a blood cell-specific gene regulatory network (GRN) by reshaping the chromatin and TF binding landscape,^16^ and (3) the repression of the endothelial GRN by the concerted action of RUNX1 and interacting co-repressors.^17^^,^^18^ If R/E is expressed prior to the EHT *in vitro*, the transition is blocked, endothelial development is abnormal, and no blood cells are formed, resembling what is found in RUNX1 knock-out mice.^19^^,^^20^^,^^21^ Consistent with this finding, the expression of R/E in the germline of mice leads to hemorrhaging at multiple sites of the developing embryo.^5^ If R/E is expressed after the EHT, it generates cell populations with enhanced self-renewal and reduced differentiation capacity.^19^^,^^20^ However, since the translocation is a clonal event *in vivo*, it is still unclear how such cell populations develop.

An important consequence of leukemogenic mutations affecting gene regulation is the derailing of differentiation resulting from compensatory cellular changes in GRNs which can result in the ectopic activation of genes unrelated to myelopoiesis or even to blood cell development in general.^22^^,^^23^^,^^24^ Each AML mutational sub-type therefore displays its own cellular identity and chromatin landscape, which in t(8;21) AML is already manifest in leukemic stem cells (LSCs). Such cells maintain expression of the fusion transcripts and are quiescent, potentially serving as a reservoir for relapse of the disease.^25^ We recently showed that t(8;21) LSCs transiently reactivate aberrant signaling pathways to give rise to rapidly proliferating blast cells.^26^ One of these ectopically activated pathways includes the gene encoding vascular endothelial growth factor alpha (VEGFA) and its receptor which is encoded by the *KDR* gene. The inhibition of VEGF-signaling significantly reduced blast numbers both *in vitro* and *in vivo*, demonstrating that it is of major relevance for leukemogenesis of t(8;21) AML and presents a possible therapeutic target.^26^ Once blast cells start to proliferate, they become dependent on signaling from hematopoietic cytokines and their receptors.^27^ Interestingly, the activation of endothelial genes is a hallmark of several leukemia subtypes driven by fusion genes involving core binding factor (reviewed in Kellaway^28^). Our study also identified a molecular circuitry consisting of R/E, RUNX1, GATA2, and the MAP kinase-responsive TF AP-1 as being responsible for LSC growth activation.^26^ However, how and when the expression of R/E activates this circuitry and how oncogene expression determines the phenotype of the cells directly after the translocation event is unknown.

To understand disease progression, it is essential to study the immediate effects of the first mutational hit on the cellular phenotype. The initial mutations, which typically affect self-renewal, are often followed by secondary mutations in genes encoding growth factor receptors that create constitutively active molecules.^29^^,^^30^ To devise new treatments, it will be necessary to understand the contribution of each type of mutation on the path to leukemogenesis. The differentiation of pluripotent stem cells (PSCs) into blood cells *in vitro* which recapitulates early hematopoietic specification including the EHT has been a valuable model to address such questions. We previously described a human PSC line which expressed an inducible version of R/E and showed that the induction of the oncoprotein led to extensive alterations of gene expression and chromatin structure.^19^ However, the precise phenotype of the first cells forced to express the R/E fusion gene was unknown.

To investigate the activation of aberrant signaling pathways resulting from R/E expression, we analyzed cells generated using an improved *in vitro* differentiation system capable of generating multi-lineage engrafting, definitive hematopoietic stem cells followed by single-cell analyses.^31^ R/E expression in myeloid precursors derived from such cells led to a (1) a dosage-dependent cell cycle block and (2) the rapid down-regulation of hematopoietic genes, both of which were associated with the loss of RUNX1 activity. Finally, R/E binding was correlated with an irreversible activation of an endothelial-like cellular phenotype. However, when R/E and RUNX1 protein expression were similar, a sub-population of myeloid blood progenitors escaped reprogramming and survived without hematopoietic cytokines when co-cultured with endothelial cells from the same cultures. Our experiments provide a mechanistic explanation for the activation of endothelial genes in t(8,21) AML and the appearance of proliferating pre-leukemic cells.

## Results

### Induction of RUNX1-ETO in ESC-derived myeloid cells leads to a dosage-dependent cell cycle block and activates an endothelial gene expression program

To dissect the molecular features of R/E expressing pre-leukemic cells, we examined the consequences of its induction in the context of our recently described a differentiation protocol based on induced pluripotent stem (iPSC) cell that generates HSC-like cells, termed iHSCs^19^^,^^31^ (Figure 1A). After 13–15 days of differentiation of the R/E expressing embryonic stem cell (ESC) line, non-adherent CD34^+^ hematopoietic stem and progenitor cells (HSPCs) were expanded for up to 8 days in a medium containing the hematopoietic cytokines SCF, TPO, IL-3, and EPO on an orbital shaker (Figure 1A). R/E was induced by adding varying concentrations of doxycycline (Dox), and cells were subsequently characterized at different time points of induction using cell proliferation assays, surface marker and gene expression analyses. Relative levels of *RUNX1-ETO* and wild-type *RUNX1* mRNA expression were determined by qPCR.

**Figure 1:**
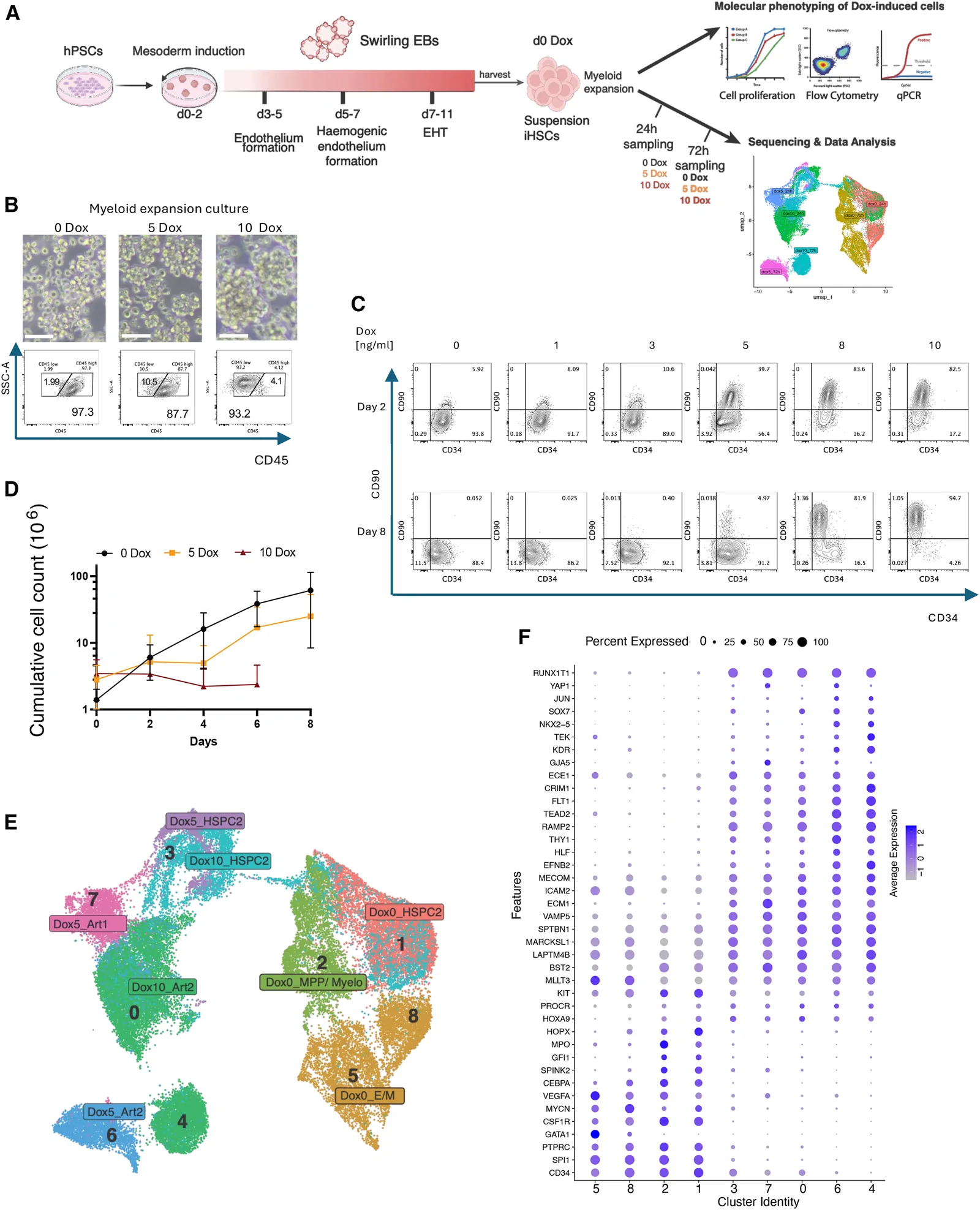
Expression of RUNX1-ETO leads to dosage-dependent arterial patterning and a cell cycle block (A) Schematic of the differentiation system showing the time course of hematopoietic differentiation from hESCs to HSPCs and further expansion after Dox induction, followed by data analysis. Partially created using BioRender.com. (B) Brightfield images of ESC-derived hematopoietic cells under no Dox (0 Dox), 5 ng/mL Dox and 10 ng/mL Dox conditions showing loss of CD45 expression (bottom). Scale bars represent 500 μm. (C) Flow cytometric analysis of CD90 and CD34 surface marker expression at day 2 and day 8 of RUNX1-ETO induction using the indicated concentrations of Dox (1, 3, 5, 8, and 10 ng/mL) compared to uninduced cells (0 Dox). (D) Box and whisker plot showing cumulative cell counts following Dox-induction (0, 5, and 10 ng/mL) conditions over 8 days. Error bars, mean ± s.e.m., *n* = 3. (E) UMAP representation of eight Seurat clusters highlighting cell populations with annotated hematopoietic and non-hematopoietic identity as identified in Figure S1 and by comparison with embryo data.^31^^,^^32^ HSPC2, hematopoietic stem and progenitor cells, embryonic cluster 2; Art1/2, arterial cells, embryonic clusters 1 and 2; Myelo, myeloid progenitor cells; E/M/M, erythroid, megakaryocyte, and mast cell progenitors. (F) Dot plot depicting the relative expression levels of arterial, hemogenic endothelium, and hematopoietic genes differentially expressed genes in Seurat clusters.

The level of R/E expression was Dox concentration dependent between days 2 and 8 of induction but did not immediately impact on the expression of *RUNX1* mRNA (Figures S1A and S1B). However, within 2 days following the initiation of Dox treatment, cells dramatically altered their phenotype, changing from non-adherent cells to cells forming large clusters (Figure 1B, middle and right). Flow cytometry analysis showed this morphological change was accompanied by an increase in the side scatter of the treated cells (Figure 1B, lower figures; Figure S1C), and coincided with loss of the hematopoietic surface marker, CD45 (Figure 1B, lower figures). At a Dox concentration of 5 ng/mL, where protein levels of RUNX1 and R/E were similar (Figure S1E),^19^ RUNX-ETO expression caused the emergence of a CD34loCD90+ cell population (Figure 1C). At higher Dox concentrations, cells displayed a dosage-dependent growth impediment (Figure 1D).

To gain insight into the nature of cells after R/E induction, we used scRNA-Seq to examine the impact of induction time (24 and 72 h) and Dox concentration (0, 5, and 10 ng/mL) on gene expression (Figure 1A). To assign cell types, we compared gene expression profiles to single-cell gene expression profiles of developing blood cells in the human embryo.^32^ This analysis had previously confirmed the HSC/HSPC phenotype of *in vitro* produced cells and identified cells of arterial origin (Art) as giving rise to the hemogenic endothelium which then generated all hematopoietic lineages.^32^ The annotation of the different cell clusters arising in the myeloid expansion medium using the expression of cluster-defining genes (Figures 1E, 1F, S1F, S1G, and S1I–S1M; Data S1) revealed the presence of an HSPC cluster (cluster 1) and a continuum of more differentiated myeloid (cluster 2) and erythroid/megakaryocytic (clusters 5 and 8) progenitor cells in uninduced cultures.

Induction of R/E led to dramatic changes in gene expression, with Dox-induced cells forming clusters distinct from untreated cells including a Dox-dependent cluster representing a subpopulation with a cell cycle block in G1 (Figures S1H and S1I). Except for cluster 3 which represented an intermediate cell type expressing a mixture of endothelial and hematopoietic genes (Figures S1H, S1I, and 1F), the majority of R/E expressing cells adopted an endothelial (arterial) phenotype, annotated Art1/Art2. Induced cells in clusters 0, 4, 6, and 7 expressed endothelial genes that were essentially absent from control cells and from clusters 1, 2, 5, and 8 consisting of HSPCs (cluster 1) and differentiating early myeloid (cluster 2) and erythroid/megakaryocytic lineages (clusters 5 and 8) (Figure 1F). Pseudotime trajectory analysis performed by using Slingshot^33^ (Figure 2A) and Monocle^34^ (Figures S2A–S2C) showed (1) that all R/E expressing endothelial-like cells originated from HSPCs (cluster 1) and (2) the reprogramming kinetics toward the arterial phenotype were Dox concentration and time dependent.

**Figure 2:**
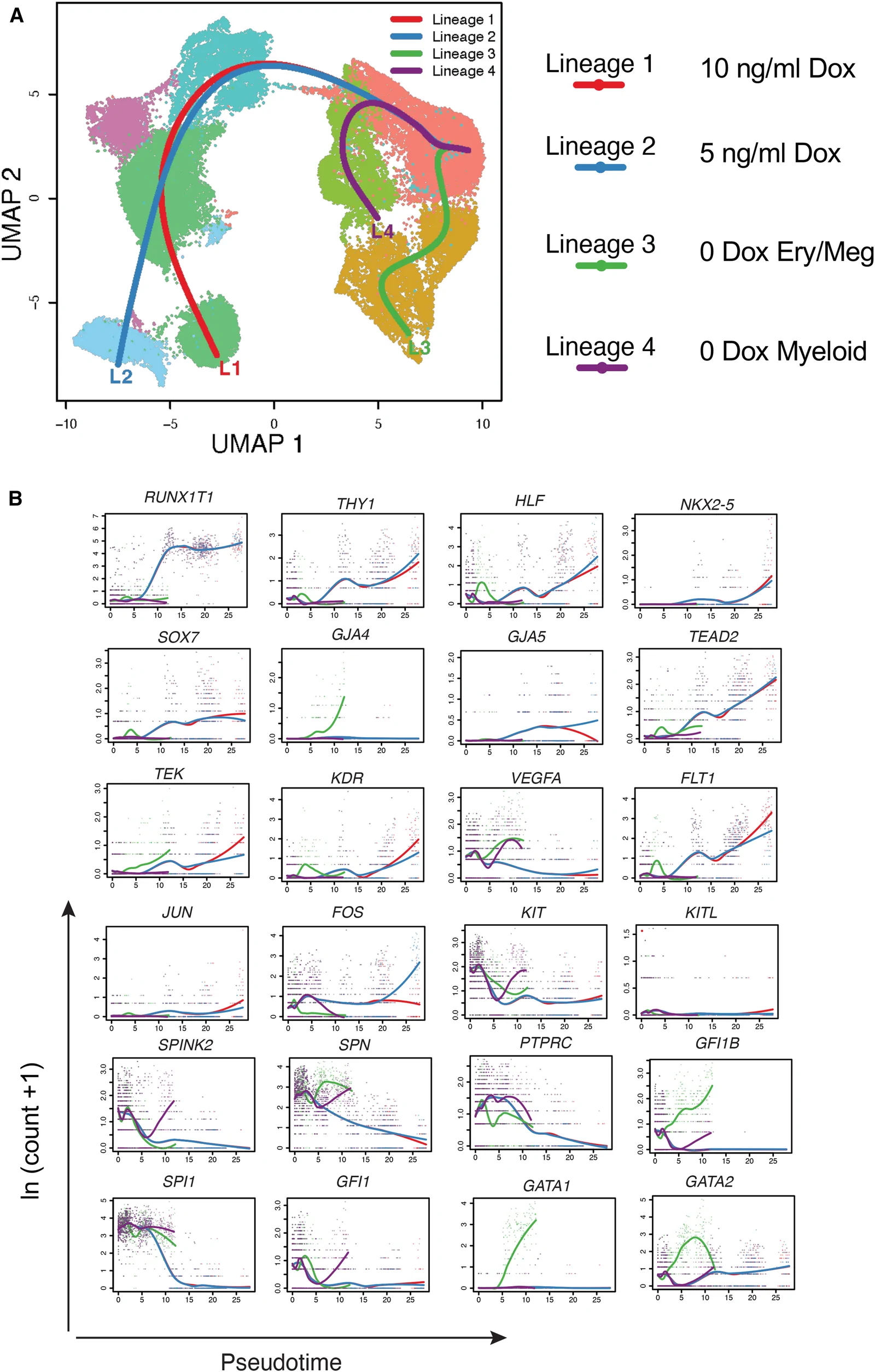
Expression of RUNX1-ETO alters the developmental control of gene expression and the differentiation trajectory (A) Projection of the differentiation trajectory of cells in our samples (0 Dox, 5 ng/mL Dox, and 10 ng/mL Dox) on the eight Seurat clusters highlighting cell populations with annotated hematopoietic and non-hematopoietic identity as in Figure 1E using Slingshot analysis defining four lineages. Lineage 1, 10 ng/mL Dox; lineage 2, 5 ng/mL Dox; lineages 3 and 4, 0 Dox. (B) Gene expression dynamics along the four trajectories, modelled using tradeSeq.

We next employed tradeSeq^34^ to measure differential expression of genes along four trajectories and examined how expression of marker genes for the different cell populations was dynamically changed during R/E induction (Figures 2B, S2D, and S2E; Data S2 and S3). This analysis showed the normal trajectory which starts with HSPCs (Figures 2A and 1E for cluster annotation) and progresses toward erythroid, megakaryocytes, and myeloid cells (lineages 3 and 4). Cells induced with 5 or 10 ng/mL of Dox show a trajectory that immediately progresses to arterial cells and, dependent on the R/E level, then splits up into two terminal arterial cell clusters (lineages 1 and 2; Figures 2A and 1E) which was also seen in the Monocle trajectory analysis (Figures S2A–S2C). When plotted against the different trajectories (1–4) detected in Figure 2A, we noticed a synchronous induction of R/E (panel RUNX1T1) in all cells treated with either 5 or 10 ng/mL Dox verifying the cell-intrinsic nature of the biological effects we were observing (Figure 2B). *k*-means clustering of co-ordinately regulated genes identified four clusters with expression profiles that were specific for cells induced with 5 ng/mL Dox (Figure S2D; Data S2) and five clusters unique to cells induced with 10 ng/mL Dox (Figure S2E; Data S3), verifying the global nature of GRN shifts we observed.

The order of reprogramming events can be deduced from Figures 2A and 2B. Here, we plotted the up- and down-regulation of specific genes according to tradeSeq analysis which indicates which genes respond immediately to R/E induction and which ones show a delayed response. These data show that the establishment of the endothelial fate is a complex process. The myeloid master regulator gene *SPI1* which is a RUNX1 and R/E target^2^ was immediately down-regulated after induction together with the blood cell-specific *SPN* gene (Figure 2B). *GATA1* was exclusively up-regulated in the erythroid/megakaryocyte (E/M) lineage in cultures not treated with Dox, accompanied by a transient up-regulation in *GATA2* expression, which was then switched off as cells matured. *GFI1* and *GFI1B* were dynamically expressed in hematopoietic cells but were silenced following R/E induction in cells with an arterial phenotype. Genes expressed in the hemogenic endothelium and endothelial genes in the induced cells such as *HLF*, *TEAD2*, *THY1*, *SOX7*, *GJA5*, the angiopoietin receptor gene *TEK*, and the VEGF receptor genes *FLT1* and *KDR* paralleled the kinetics of up-regulation of R/E, suggesting a direct impact of the oncoprotein on their expression. Interestingly, their up-regulation followed a biphasic trajectory, suggesting that the cells underwent an initial direct reprogramming phase and then responded to indirect events before they settled on the final endothelial phase characterized by increased up-regulation of genes such as *TEK* and *KDR.* An interesting Dox dose-dependent expression pattern was seen in arterial cells at the end of each trajectory, especially with the gene encoding the AP-1 component, *FOS*.^35^ AP-1 mediates MAP kinase signaling and mediates VEGF signaling via the VEGF receptors.^26^^,^^36^ We observed a clear correlation of *FOS* expression levels with R/E induction levels, highlighting a difference in signaling responsiveness when RUNX1 and R/E are expressed at the same or at disparate levels.

### Induction of RUNX1-ETO perturbs the RUNX1 GRN

We next wished to explore how binding of RUNX1 and R/E to chromatin affected the expression of genes associated with RUNX1 binding sites to determine which cell populations were affected. RUNX1 can act as both a repressor of endothelial genes and as an activator of hematopoietic genes^17^^,^^37^^,^^38^ while R/E has been shown to recruit co-repressors leading to repression of gene expression in transient transfection assays.^2^^,^^39^^,^^40^ We first determined the pattern and the dynamics of RUNX1 and R/E binding using our previously published ChIP data from ESC-derived hematopoietic progenitor cells representing a mixed population of immature and more differentiated HSPCs which were either left uninduced or were treated with 5 ng/mL Dox.^19^ Examples of the dynamics of RUNX1 and R/E binding at specific genes are shown in Figures 3A, S3A, and S3B. The *KIT* gene is a RUNX1 target whose expression is down-regulated in endothelial cells after R/E induction. This reduction in expression coincides with a loss of RUNX1 binding at an intronic enhancer and the binding R/E to the promoter, leading to a reduction of RUNX1 binding (Figure 3A). Similarly, expression of *VAV1*, which shows RUNX1 binding at promoter and distal elements, is also diminished by R/E binding (Figure S3A). The endothelial *VEGFA* gene is bound by RUNX1 in uninduced cells, and RUNX1 binding is reduced after binding of R/E at the same sites after induction (Figure S3B).

**Figure 3:**
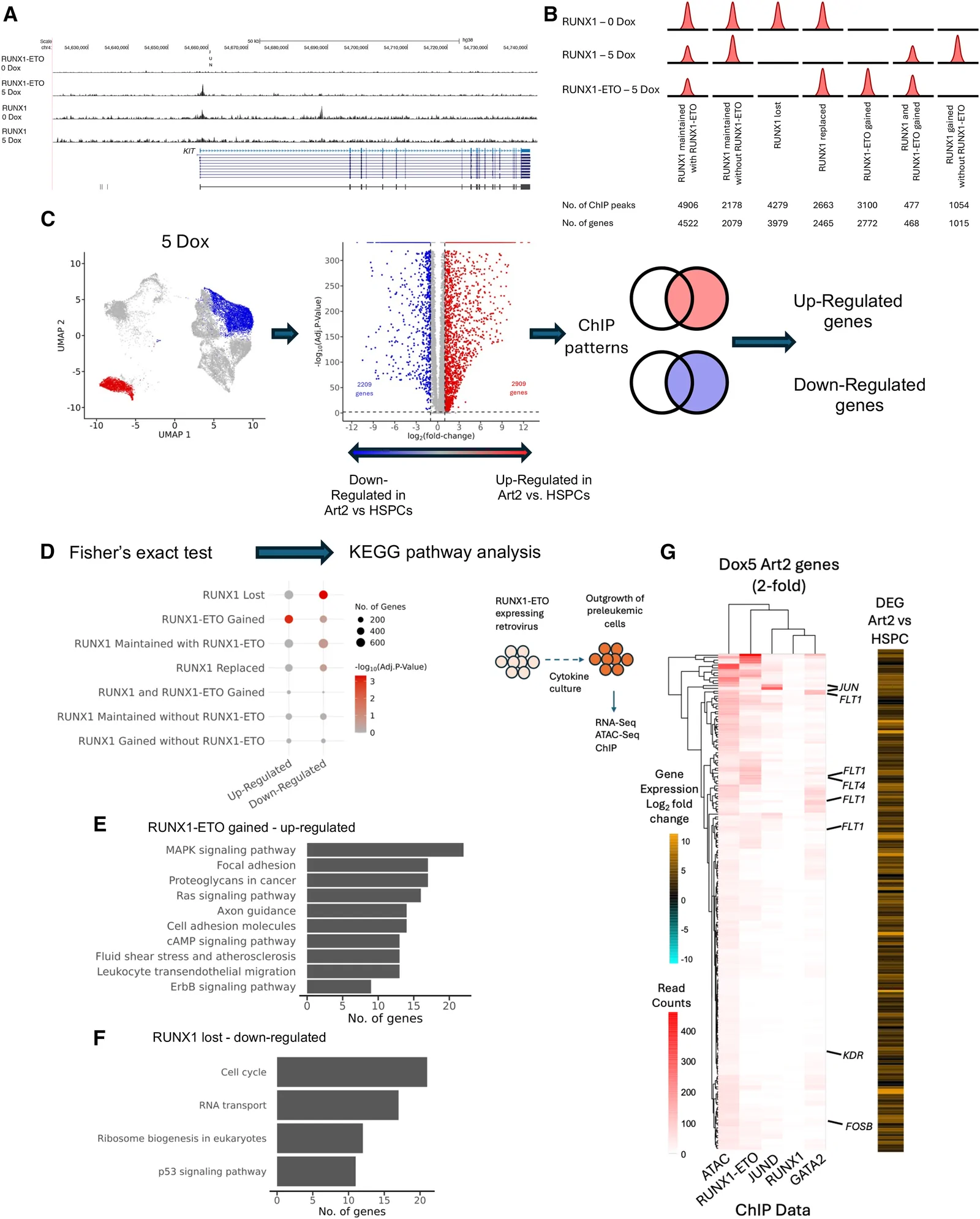
RUNX1-ETO interferes with the RUNX1-driven GRN (A) UCSC browser screenshots showing RUNX1 and RUNX1-ETO binding to the *KIT* locus in the presence or absence of 5 ng/mL Dox. (B) Depiction of seven different classes on RUNX1 and RUNX1-ETO binding patterns obtained from previously conducted ChiP experiments using the same cell line^19^ from uninduced or 5 ng/mL Dox-induced HSPCs. Peaks were filtered against ATAC-Seq peaks. Shared peaks are depicted with half the height. The table below summarizes peak numbers per group. Genes associated with binding sites as determined by comparison with promoter-capture HiC data from human CD34^+^ cells^41^ are listed in the bottom line. (C) Strategy of determining up- and down-regulated RUNX1 and RUNX1-ETO target genes in the 5 ng/mL Dox-induced Art2 cluster (red) as compared to that of the uninduced HSPC2 cluster (blue). Left: UMAP of 0 and 5 ng/mL Dox cells highlighting the HSPC and Art2 cell populations at the start and end of the differentiation trajectory. Right: volcano plot of genes which are differentially expressed between HSPC and Art2 cell populations in 0 and 5 ng/mL Dox cells. A gene was considered differentially expressed if it had a 2-fold change and an adjusted *p* value less than 0.01. Right: Integration of differential gene expression values with the different patterns of RUNX1 and RUNX1-ETO binding as depicted in (B). (D) Results from a one-sided (right-tailed) Fisher’s exact test comparing the overlap of genes up- and down-regulated in Art2 vs. HSPCs to ChIP binding groups depicted in Figure 2B. Gene sets with a Benjamini-Hochberg corrected *p* value < 0.05 were considered significant. (E and F) KEGG pathway enrichment of significant gene sets in this case up-regulated genes gaining RUNX1-ETO binding (E) and down-regulated genes that have lost RUNX1 binding. Lists of enriched genes can be found in Data S5. (G) Right: differentially expressed Art2 genes (2-fold change against HSPCs) were matched with the corresponding DHS from the RUNX1-ETO expressing cord blood cells from Chin et al. as depicted in the scheme on the left.^27^ Left: raw counts for the ATAC and ChIP (RUNX1-ETO, JUND, RUNX1, and GATA2) data from the cord blood cells were aligned against each other and clustered.

Our global analysis shows that after induction 2,178 RUNX1 peaks were maintained but most sites either lost RUNX1 binding or binding was diminished by R/E binding to the same sites (Figure 3B). We also counted 1,054 RUNX1-only peaks, 4,096 RUNX1 peaks shared peaks with R/E, and 3,100 R/E-only peaks. To assign binding sites to genes, we used published HiC data for human hematopoietic progenitor cells^22^^,^^42^ (Figure 3B, bottom) and determined which genes were differentially expressed between cells from control versus Dox-induced cultures. We compared gene expression within cells representing cluster 1 (control) to gene expression within clusters 4 (5 ng/mL Dox) and 6 (10 ng/mL Dox) (Figures 3C and S3C, middle figures) representing endothelial cells (Art2). To gain an insight into the regulatory mechanisms underpinning the observed changes in gene expression, this analysis was integrated with the ChIP data. Using a Fisher’s exact statistical test, we investigated whether changes in expression of certain groups of genes were associated with a specific binding behavior of RUNX1/R/E (Figures 3D and S3D; Data S5). This analysis revealed that the up-regulation of genes in endothelial cells was correlated with the overall gain of R/E binding at these genes, while the down-regulation of genes in these cells correlated with the loss of RUNX1 binding as compared to HSPCs. Importantly, KEGG pathway analysis revealed that both enriched groups of genes belonged to different pathways (Figures 3E, 3F, S3E, and S3F). The loss of RUNX1 affected the expression of cell cycle and ribosome biogenesis genes (Figure 3E; Data S5), which links this binding pattern to the loss of proliferation. R/E bound to up-regulated genes of the MAP-kinase/RAS signaling and focal adhesion/angiogenesis pathway such as *JUN*, *FOS*, *FYN* as well as *VAV3* and *ITGA9* (Figures 3F and S3F; Data S5), linking this binding pattern to the activation of the endothelial phenotype.

In a previous study, we transduced human CD34^+^ cord blood progenitor cells with a R/E expressing retrovirus.^27^ We examined gene expression changes and the chromatin structure of transduced and un-transduced cells together with the binding pattern of R/E, RUNX1, GATA2, and AP-1. To test whether the activation of endothelial genes was also observed in this setting, we identified 2-fold up-regulated genes in ESC-derived Art2 cells and associated them with their respective *cis*-regulatory elements as described in Figure 3C. We next correlated such sites with differentially activated open chromatin regions from R/E-transduced CD34^+^ cord blood cells as well as with the TFs binding to such sites after transduction as measured by ChIP (Figure 3G). This analysis showed that the DHS for these genes were also differentially accessible in the R/E expressing cord blood cells and were mostly bound by R/E but not RUNX1. Moreover, a subset of these genes was bound by AP-1 and GATA2 as we have shown previously.^26^ The most up-regulated genes were the AP-1 family members *JUN* and *FOSB*. The comparison of individual genes up-regulated in both transduced primary and ESC-derived R/E expressing cells (Figure 3G) as well as in primary cells from t(8;21) patients (Figure S3H and data from Assi et al.^22^ and Chin et al.^27^) revealed the common up-regulation of multiple endothelial genes such as *KDR*, *FLT1*, *VEGFA*, and *TEK*.

We have previously shown that the R/E binding pattern observed in ESC-derived progenitor cells resembled that found in t(8;21) cell lines.^19^ To see whether this was also true for primary cells from t(8;21) patients, we compared our previously published ChIP data from a t(8;21) patient^2^ with those of ESC-derived Dox-induced cells and transduced CD34^+^ cord blood cells (Figure S3H). Despite the different quality of the data, when ranked against each other we observed the same general binding pattern. Taken together, our analyses show that the activation of an endothelial program is associated with oncogene expression and a specific oncoprotein binding pattern in multiple cellular settings.

### Induction of RUNX1-ETO blocks hematopoietic differentiation in a dose-dependent fashion

We next studied how R/E induction affected the differentiation of hematopoietic cells, and whether the withdrawal of the Dox inducer restored the normal phenotype. To this end, we performed methylcellulose (MC) colony-forming assays in the presence or absence of Dox as depicted in Figure 4A. The R/E inducible PSC line carries a reporter for the *SOX17* gene which is expressed in arterial and hemogenic endothelia. Removal of Dox from the medium caused a rapid down-regulation of R/E mRNA expression (Figure S4A) but did not significantly affect the expression of *SOX17* (Figure S4B). We then tested (1) whether the timing and duration of Dox treatment impacted the ability of cells form colonies and (2) whether these variables affected the nature of the colonies that were formed (Figures 4B–4E). Examples of the different colony types are shown in Figure S4D. After 2 days of further culture (day 15) in the absence of Dox (0 Dox), HSPCs generated a similar frequency and spectrum of CFU-GEMM (colony forming unit granulocyte, erythrocyte, macrophage, megakaryocyte), CFU-GM (granulocyte, macrophage), and CFU-G (granulocyte) colonies (Figures 4B and 4C). Treatment of cells with 5 ng/mL Dox for 2 days prior to the initiation of colony forming assays did not significantly reduce the total colony number in MC cultures without Dox (5 Dox-Disc). However, maintaining Dox led to a significant reduction in colony numbers (5 Dox-Cont) (Figures 4B and S4F), most likely resulting from the observed cell cycle block. A more pronounced effect was observed when cells were treated with 10 ng/mL Dox prior to MC assay initiation, with a significant reduction in the number of CFUs (10 Dox-Disc) in MC cultures lacking Dox and the complete absence of CFUs when Dox was maintained (10 Dox-Cont) (Figures 4B, 4C, and S4F). Cells from cultures grown for 8 days the absence of Dox (0 Dox) generated no CFUs at all, suggesting that they had differentiated beyond the clonogenic progenitor stage (Figures 4D and 4E). In contrast, HSPCs treated with 5 ng/mL Dox for 8 days prior to seeding retained colony forming capacity, irrespective of whether the Dox treatment was continued (5 Dox-Cont) or withdrawn (5 Dox-Disc) (Figures 4D and 4E). This retention of colony forming ability is consistent with R/E expression impairing myeloid differentiation. Under conditions in which R/E expression was continued, no mature macrophage colonies (CFU-M) formed in contrast to those where Dox was discontinued. Similar to what was observed after 2-day expansion, clonogenic cells were seen in 8-day expansion cultures when 10 ng/mL Dox was withdrawn prior to MC culture (Figures 4D and 4E). However, while the proportion (%) of specific colony types was similar, the absolute number of colonies was greatly diminished (Figure 4D).

**Figure 4:**
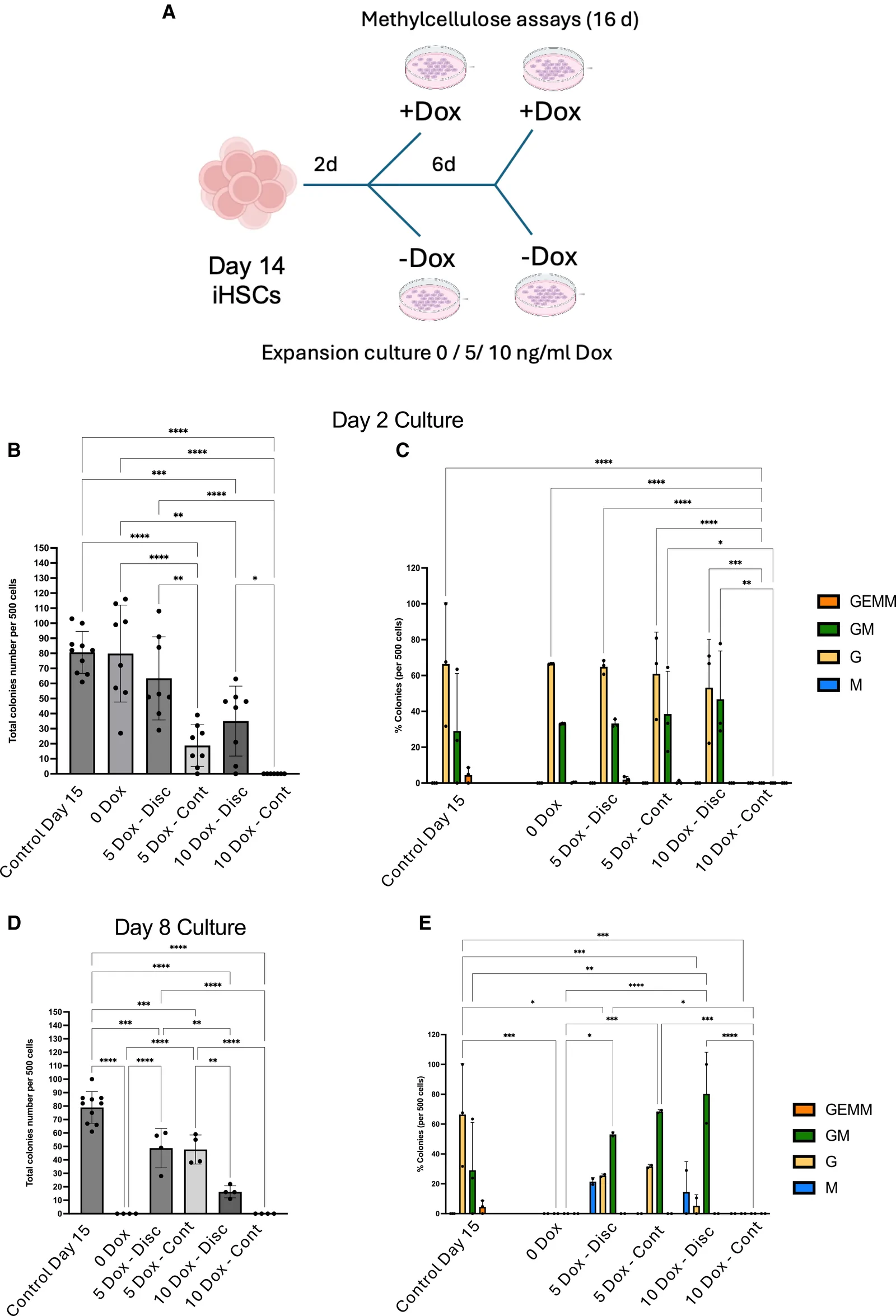
Induction of RUNX1-ETO interferes with hematopoietic colony formation, proliferation, and differentiation (A) Scheme of colony assays. Partially created using BioRender.com. (B) Total number of colonies per 500 cells plated at day 2 of Dox culture. 0 Dox, uninduced cultures; 5 Dox-Disc, 5 ng/mL Dox induction and Dox withdrawn from MC; 5 Dox-Cont, 5 ng/mL Dox induction and Dox added to MC; 10 Dox-Disc, 10 ng/mL Dox induction and Dox withdrawn from MC; 10 Dox-Cont, 10 ng/mL Dox induction and Dox added to MC. (C) Colony types quantified as CFU-GEMM (granulocyte, erythrocyte, monocyte, megakaryocyte), CFU-GM (granulocyte/macrophage), CFU-G (granulocyte), and CFU-M (macrophage), expressed as a percentage of total colonies found in (B). (D and E) Same as (B) and (C) but following 6 days of culture in expansion medium before MC plating. Bars represent mean ± SD from *n* = 3 independent experiments. Statistical significance was assessed using an ordinary one-way ANOVA (B, D) or a two-way ANOVA (C, E) followed by Tukey’s multiple comparisons test. ^∗^*p* < 0.05; ^∗∗^*p* < 0.01; ^∗∗∗^*p* < 0.001; ^∗∗∗∗^*p* < 0.0001.

### Induction of RUNX1-ETO generates endothelial cells in a dose-dependent fashion

While the induction of a gene expression program resembling that of arterial cells was an intriguing indication that iPSC-derived HSPCs adopted an endothelial identity, we wished to test whether they displayed other endothelial features. We therefore differentiated day 14 HSPCs in a myeloid expansion medium with or without 5 or 10 ng/mL Dox. On day 2 of expansion, induced cells were flow cytometrically sorted to enrich for CD90^+^CD34^+^ surface expression (Figure 5A). Uninduced cells were sorted based on CD34 and CD45 expression, because they lacked surface CD90 (see Figure 1C). Sorted cells were re-cultured in fresh medium with the same growth factors. At day 6, we cultured uninduced cells and induced cells in a Matrigel-based endothelial network assay in the presence of VEGF (Figures 5B–5F) but no hematopoietic cytokines. Quantitative analyses of these experiments together with examination of additional surface markers are shown in Figures 5G, 5H, and S5C–S5F. Inspection of the cells after 2 days in Matrigel culture revealed a Dox-dependent alteration in cell shape and surface marker expression as compared to uninduced cells (Figures 5B, 5C, and 5E, left figures). Cells started to adhere, interact, and form a network of tubes. These changes were accompanied by the up-regulation of endothelial markers on the cell surface (CD90 and CD201) (Figures 5B, 5C, and 5E, right figures). Again, the proportion of cells undergoing these changes was dosage dependent. Cultures of cells induced with 5 ng/mL Dox contained a mixture of hematopoietic and endothelial cells (Figure 5C) whereas 10 ng/mL Dox cells contained few residual blood cells (Figure 5E). Confocal microscopic examination of cells in Matrigel cultures show a striking, dose-dependent difference in cell morphology and composition: 5 ng/mL Dox cultures contained the expected mixture of SOX17-mCHERRY expressing endothelial and non-expressing hematopoietic cells (Figure S5G) whereas 10 ng/mL Dox cultures displayed no hematopoietic cells but contained exclusively aggregated clusters of SOX17 expressing cells starting to form network connections (Figure 5I).

**Figure 5:**
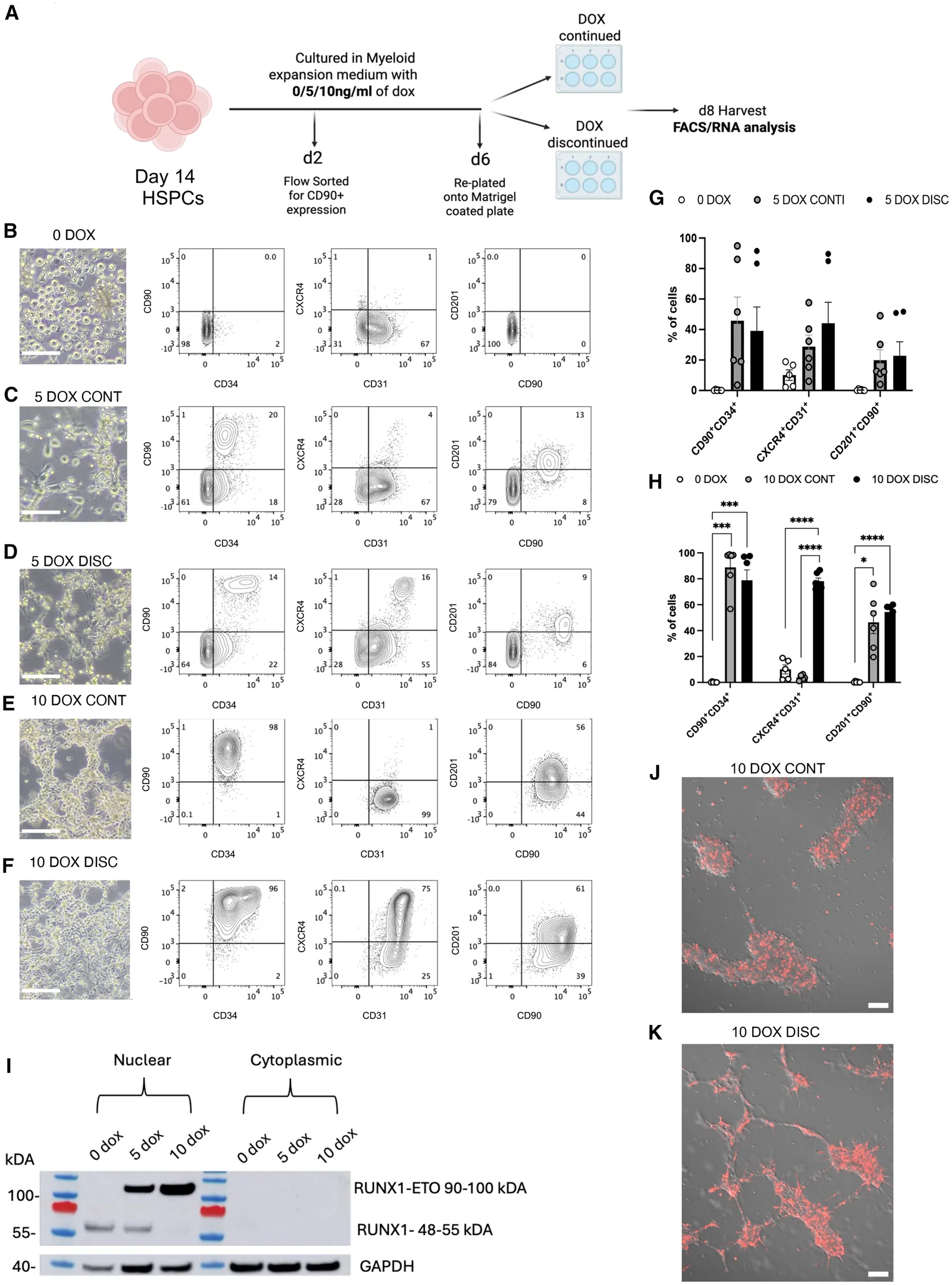
Induction of RUNX1-ETO leads to the generation of endothelial cells in a dose-dependent and irreversible fashion (A) Schematic of endothelial reprogramming and withdrawal experiments followed by Matrigel culture (MC) containing 2 ng/mL BMP, 5 ng/mL IGF1, 5 ng/mL rh IGF2, 10 ng/mL FGF2, 5 ng/mL EGF, and 25 ng/mL VEGF. After a total of 8 days, cells were harvested for flow cytometry and transcriptomic analysis. Partially created using BioRender.com. (B–F) Images (left) and flow cytometry analyses of cells after a total of 8 days culture (see A) with the indicated surface markers. (B) Uninduced cells (0 Dox). (C) 5 Dox-Cont: 5 ng/mL Dox induction and Dox continued in MC. (D) 5 Dox-Disc: 5 ng/mL Dox induction and Dox discontinued in MC. (E) 10 Dox-Cont, 10 ng/mL Dox induction and Dox continued in MC. (F) 10 Dox-Disc, 10 ng/mL Dox induction and Dox discontinued in MC. Representative analyses of *n* = 6 experiments are shown. (G and H) Bar graphs of flow cytometry data for the indicated surface markers in cells treated as shown in (A). Error bars, mean ± S.E.M., *n* = 6 experiments. (I) Western blot of nuclear and cytoplasmic extracts of HSPCs following 4 days of expansion in 0 Dox, 5 ng/mL Dox, or 10 ng/mL Dox. An anti-AML1 (55 kDA) antibody detecting the RUNX1 RUNT domain was used to detect RUNX-ETO (100 kDA) fusion protein and endogenous RUNX1 (55 kDA) protein levels. GAPDH (36 kDa) was used as a loading control. (J and K) Confocal images of representative Matrigel culture cultured in 10 ng/mL Dox (J, Cont) or where Dox was withdrawn (K, Disc) with the red color coming from the SOX17 reporter expressing mCherry. Scale bars represent 100 μm.

### RUNX1-ETO-mediated endothelial reprogramming is irreversible

Our phenotypic analysis showed that the level of R/E expression affected the growth and the proportion of hematopoietic cells and endothelial cells after induction. R/E has been identified as a potential therapeutic target for siRNA therapy as leukemic cells have been shown to differentiate after its mRNA was depleted, demonstrating that even in the context of multiple mutations the presence of the oncoprotein was essential for leukemogenesis.^2^^,^^4^^,^^43^ However, R/E can recruit co-regulators such as histone deacetylases.^39^^,^^40^ Our phenotypic analysis therefore did not exclude the possibility of persisting, heritable gene expression changes. We therefore withdrew Dox from the induced cells and measured surface marker expression as described in Figure 5A. The quantitative analysis of FACS profiles (Figures 5D, 5F–5H, S5D, and S5F) demonstrated that R/E induction caused a profound further shift of cellular identity from a hematopoietic toward an endothelial phenotype which was particularly striking with 10 ng/mL Dox. The percentage of remaining CD45+SSC^low^ hematopoietic cells in the culture was dosage dependent and did not revert after Dox withdrawal, indicating that the induced endothelial phenotype was irreversible (Figure S5B). Moreover, once R/E was withdrawn, cells moved closer to an endothelial phenotype by up-regulating the endothelial markers CXCR4 and CD31 (Figures 5D–5F, right figures). FACS staining (Figure S5I) showed that these cells also up-regulated the NOTCH ligand DLL4 which is essential for angiogenesis and artery development,^44^ further enhancing the endothelial phenotype. *SOX17* mRNA expression persisted upon withdrawal, suggesting that once cells had committed to the endothelial fate, it was irreversible (Figure S4B). The same result was seen by confocal microscopy (Figures 5J and S5H). SOX17 reporter expressing cells in 10 ng/mL Dox cultures started to form networks of tube-like connections, again suggesting that they acquired more endothelial characteristics. We next sought to explore the reason for this result by measuring RUNX1 protein expression as this TF is absolutely required for hematopoietic development and its protein stability is under cell cycle-dependent post-translational control.^45^ Western blot analysis showed a complete lack of RUNX1 protein in 10 ng/mL Dox cultures providing a molecular explanation for the absence of hematopoietic development (Figure 5K).

Taken together, our data show that (1) R/E blocks hematopoietic differentiation which resumes after withdrawal of Dox, (2) once endothelial cells are formed, the withdrawal of the oncoprotein does not reverse their phenotype correlating with the absence of RUNX1 protein expression, (3) R/E induction affects differentiation of an early multipotent progenitor cell prior to the CFU-GM stage, which is in accordance with the finding that the viability of myeloid cells is no longer RUNX1 dependent,^46^ and (4) when expression of RUNX1/R/E is similar, a subpopulation of induced cells retains hematopoietic potential.

### RUNX1-ETO expressing myeloid progenitors survive when cultured with endothelial cells

We wanted to gain more insight into the gene expression of cells in the Matrigel cultures in which Dox was continued or withdrawn (Figure 4F). Initial qPCR assays examining the expression of endothelial and hematopoietic genes in these mixed populations confirmed the dynamic response to Dox withdrawal seen by flow cytometry. Increased *DLL4* and *CXCR4* expression was seen in cultures where Dox was discontinued and indicated arterial patterning, but these analyses did not inform which cells responded (Figure S4C). We therefore explored whether 5 ng/mL Dox-induced cells retained an imprint of R/E oncogene exposure and performed scRNA-Seq analyses of four samples: the starting cell population (day 14 HSPCs), day 22 Matrigel cultures containing uninduced control cells, and day 22 Matrigel cultures with and without 5 ng/mL Dox for the last 2 days of culture (Figure 5A). The composite analysis of all four samples showed that uninduced day 14 and day 22 populations clustered separately, but that the continued and withdrawn day 22 cells clustered as one large population in addition to two distinct arterial populations (Figure S6A). Cell type classification yielded 13 clusters containing hematopoietic and arterial cells (Figures S6B, S6C, and S7A; Data S6).

Individual cluster analysis showed that all uninduced cells had differentiated from HSPCs to granulocytes by day 22 (Figures 6A and 6B; Data S7; Figures S6C). In contrast, induced cells still contained a significant proportion of myeloid progenitors (Myelo) (clusters 0 and 6 in Figures 6C and S6F), again demonstrating that R/E expression impaired myelopoiesis and cells were retained at an immature state. Uninduced day 14 cell populations contained a higher proportion of proliferating cells compared to uninduced day 22 cells, consistent with their more mature differentiation state (Figure S6D). In addition, this analysis together with the analysis of marker genes (Figure S6D; Data S8) confirmed that clusters 8 and 13 in Figure S6B were arterial endothelium type cells (Art).

**Figure 6:**
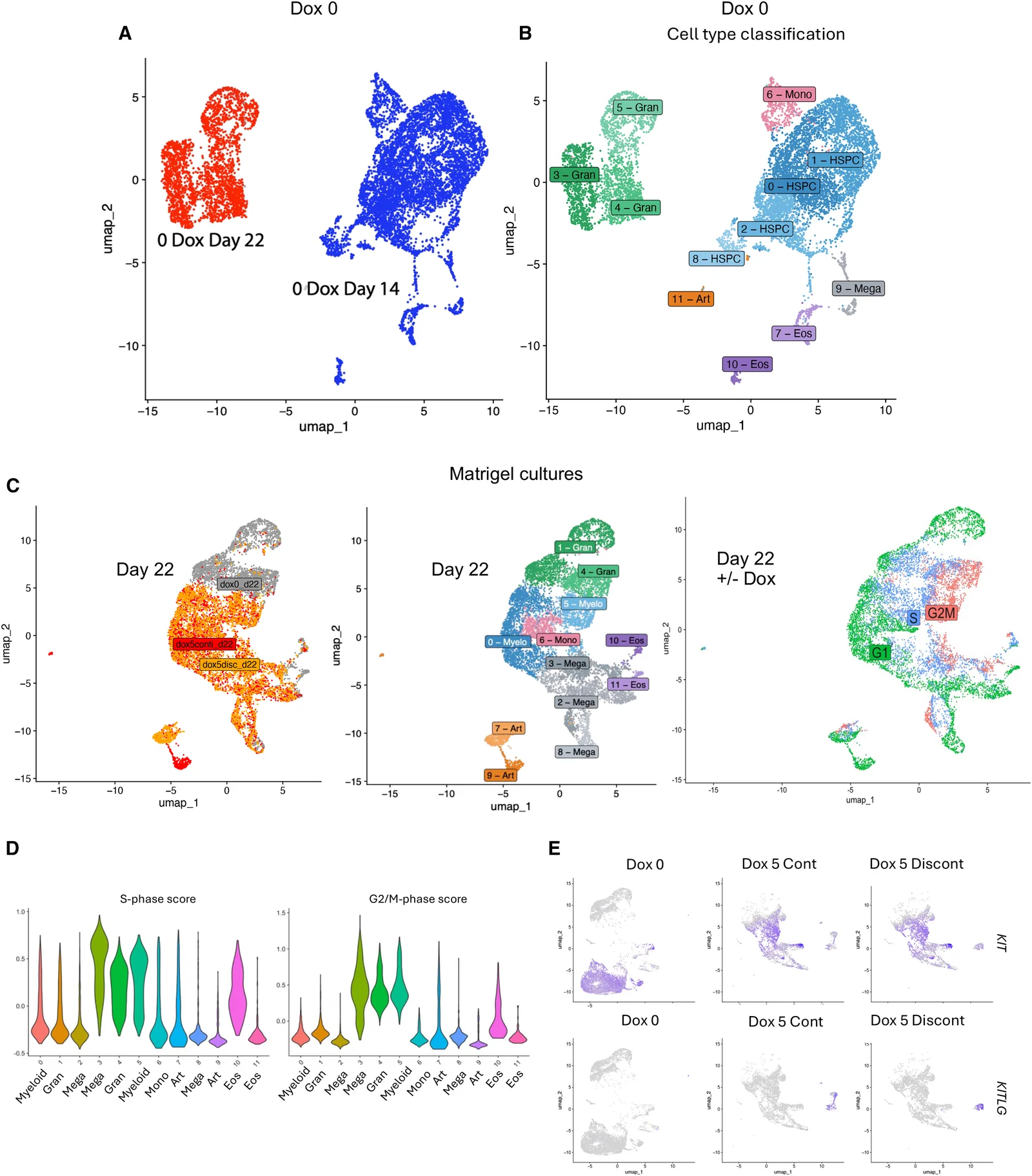
Matrigel cultures contain proliferating hematopoietic cells (A) UMAP representation of scRNA-Seq data from uninduced (Dox 0) cells at day 14 and day 22. (B) Cell lineage annotation of uninduced cells at day 14 and day 22 to 11 clusters. HSPCs, hematopoietic stem and progenitor cells; Art, arterial endothelial cells; Gran, granulocytic cells at different stages of maturation; Eos, eosinophils; Mega, megakaryocytes; Mono, monocytes. (C) Left: UMAP representation of scRNA-Seq data from day 22 cells from Matrigel cultures that were uninduced or induced with 5 ng/mL Dox, followed by Dox continuation (Dox5conti) or discontinuation (Dox5disc) for the final 2 days of Matrigel culture (see Figure 4A). Middle: cell lineage annotation of clusters of Dox treated and untreated (discontinued) cells shown in (C) showing 11 clusters. Right: UMAP representation of the cell cycle status of Dox treated and untreated day 22 cells. (D) S-phase and G2M-scores of Dox treated and untreated clusters of day 22 cells from (C). (E) Feature plots from the same cell populations as in (C) separated by their induction status (Dox 0: no induction), continued induction with 5 ng/mL Dox (Dox 5 Cont) and after Dox withdrawal (Dox 5 Discont) showing the expression levels of *KIT* and *KITLG.*

We next directly compared data from day 22 Matrigel cultured cells induced for 6 days with 5 ng/mL Dox which then was continued or withdrawn for the final 2 days (Figures S7A and S7B; Data S9). We found little difference in gene expression between the different clusters with or without Dox which was also seen when feature plots of individual genes were examined (Figure S7C). The exception were the arterial clusters 7 and 9 in Figure 6C, with cluster 9 representing 5 ng/mL Dox cells, and cluster 7 representing cells where Dox was withdrawn for the last 2 days. Closer inspection of differentially expressed genes in these two cell populations (Figure S7B) showed multiple up-regulated endothelial genes after Dox withdrawal, including *CD34*, *CDH5*, and *CXCR4,* a result which was confirmed by flow cytometry (Figures S5B–S5F, S5D, and S5F). In addition, a number of genes relevant for vascular biology such as the mechano-sensor *PIEZO2*^47^ or *ROBO4* both of which are important for endothelial barrier function were up-regulated.^48^ This analysis was confirmed by directly comparing scRNA-seq data from uninduced arterial cells with day 22 induced arterial cells split by Dox condition (Figures S7D and S7E; Data S10). The comparison highlighted up-regulation of hundreds of genes after Dox removal (Figure S7E). When characterized by Gene Ontology (GO) terms, the top enriched terms were vasculogenesis and angiogenesis (Figure S7F), demonstrating that after Dox withdrawal, cells progressed further toward endothelial differentiation without regaining hematopoietic potential.

An interesting finding was that although the Matrigel cultures (Figures 4F–4K) did not include any added hematopoietic cytokines, 5 ng/mL Dox-induced cultures contained a large proportion of hematopoietic cells even in the presence of R/E. This group of cells consisted of myeloid precursors, granulocytes, eosinophils, and megakaryocytes (Figure 6C, middle; Figure S6F). Furthermore, many cells were proliferating (Figure S6C) with a high S-phase score (Figure S6D). In addition, except for the arterial cells in clusters 7 and 9, we saw little or no difference in the gene expression profile in these cells (Figure 6C, left) in the presence and absence of Dox. This finding contrasted with the original 10 ng/mL Dox induction cultures which saw a large proportion of cells being reprogrammed and undergoing a cell cycle block, even in the presence of hematopoietic cytokines when the expression level of R/E exceeded that of RUNX1 (Figures 1D and 4F–4K). However, when R/E and RUNX1 levels were similar a subset of hematopoietic cells continued to proliferate (Figure 1D).

### Reprogrammed endothelial cells support the survival of hematopoietic cells

Endothelial cells are known to produce hematopoietic cytokines such as SCF (encoded by the *KITLG* gene).^49^ Figure 5E shows that 5 ng/mL Dox continued and withdrawn hematopoietic cells expressed the SCF receptor, KIT, but did not express *KITLG*. However, R/E induction and the development of arterial cells (clusters 7 and 9) led to expression of this gene which was further up-regulated when Dox was withdrawn (Data S9) suggesting that endothelial cells could provide growth support for co-cultured hematopoietic cells. In addition, proliferating R/E-induced cells express the VEGF receptor gene KDR (Figure S1L), which in VEGF containing cultures can kick-start the growth of blast cells from LSCs, as seen in t(8;21) patients.^26^ We therefore investigated the role of the two cytokines in sustaining the growth of hematopoietic cells as outlined in Figure 7A. We first induced day 15 HSPCs with 5 ng/mL Dox for 6 days, placed them into liquid Matrigel medium for 4 days with or without VEGF, SCF, or both, followed by measuring cell numbers (Figure S6G) and viability (Figure S6H). We also employed a KIT inhibitor, Ripretinib, which blocks signal transduction by SCF and KIT internalization.^50^ This inhibitor has so far mostly been used in gastrointestinal tumors.^51^ Cells rapidly lost viability when cultured in the absence of both cytokines. The addition of VEGF alone did not rescue proliferation/viability, but cells started to grow once SCF was added. Ripretinib blocked SCF-induced growth and KIT internalization, demonstrating that cells were not only responsive, but also dependent on SCF.

**Figure 7:**
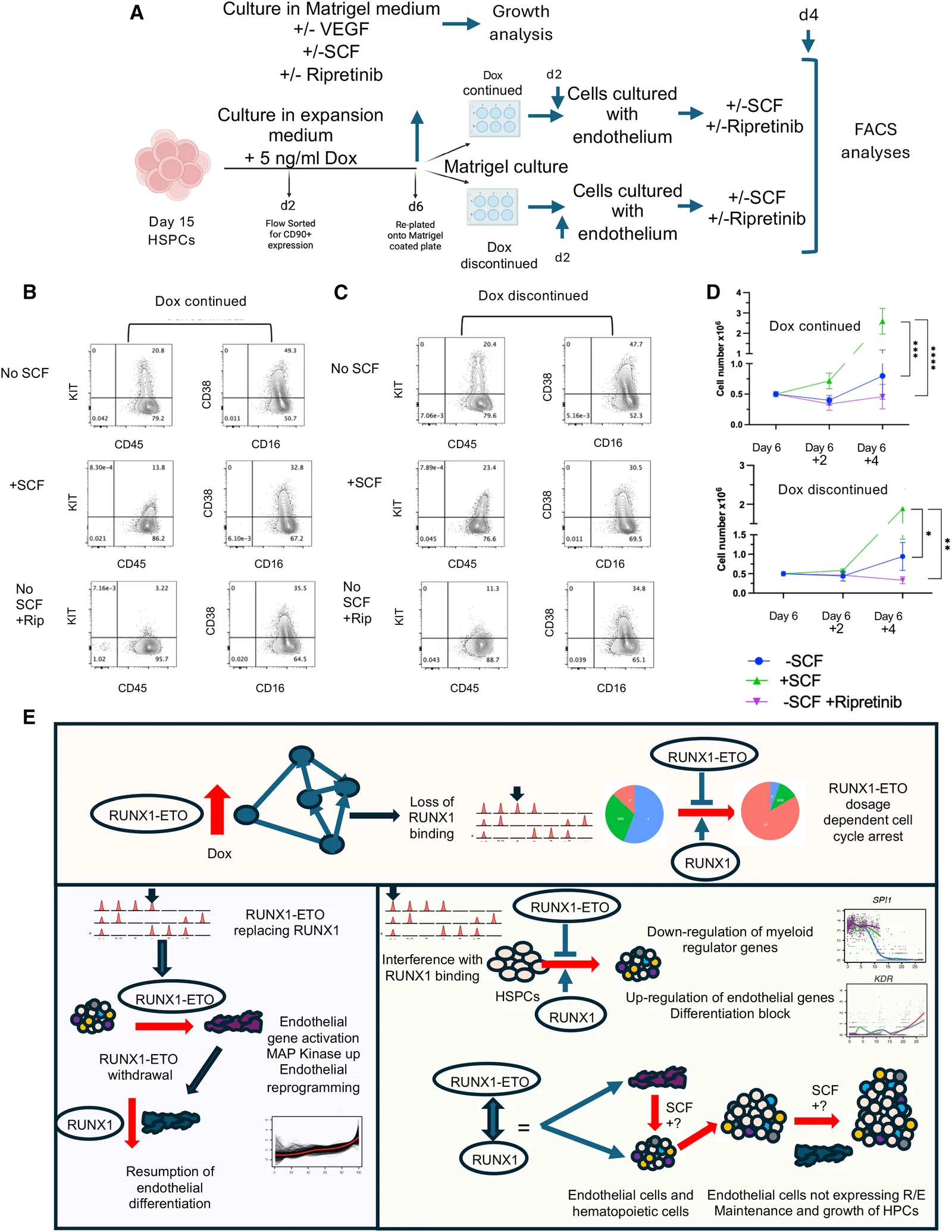
Endothelial cells support the survival and growth of hematopoietic cells (A) Scheme of experiments aimed at examining the role of cytokines (SCF and VEGF) sustaining the growth/survival of hematopoietic cells in Matrigel culture. (B and C) FACS analyses examining the proportion of KIT+, CD45^+^, CD16^+^, and CD68^+^ cells in Matrigel cultures without SCF, 100 ng/mL SCF or with 64 μM Ripretinib as indicated in cells cultured with (B) or without (C) 5 ng/mL Dox for 4 days. The panels show representatives of 3 independent experiments. (D) Cell numbers measured using propidium iodide (PI) during 4 days of Matrigel culture without SCF, with 100 ng/mL SCF or with 64 μM Ripretinib in cells cultured with (top) or without (bottom) 5 ng/mL Dox for 4 days. Data represent mean ± SEM of three independent experiments. Statistical significance assessed using two-way ANOVA with Tukey’s multiple comparisons test, ^∗^*p* < 0.03, ^∗∗^*p* < 0.002, ^∗∗∗^*p* < 0.0002, ^∗∗∗∗^*p* < 0.0001. (E) Model depicting the different steps of how RUNX1-ETO expression deregulates the GRN of myeloid progenitor cells and interferes with RUNX1 binding.

We next examined the potential growth/survival supporting role of SCF by culturing induced cells with or without Dox in Matrigel culture containing endothelial and hematopoietic cells (Figure 7A). We first added SCF and measured the number of KIT+/CD45+ cells using FACS (Figures 7B and 7C, left figures; Figure S7J) together with the surface expression of the mature myeloid markers CD16 and CD38 (right figures). Measurements of cell numbers demonstrated that in the absence of SCF, cells displayed low level proliferation (Figure 7D). Adding SCF increased cell proliferation and also caused a reduction of KIT on the cell surface as also seen in Figure S6G, but caused only a small change in the proportion of mature myeloid cells. In contrast, after addition of Ripretinib, cells completely ceased growth and cultures lost KIT+/CD45+ cells, again in cells grown with or without Dox.

Taken together, our data show that R/E expressed at high levels acts as a roadblock to both blood cell division and differentiation, diverting cells toward an irreversible endothelial fate. However, terminal endothelial differentiation was only permitted once R/E was removed. Finally, in the presence similar levels of RUNX1 and R/E, blood cells harnessed growth factors provided by endothelial cells in a co-culture setting to survive and begin to grow.

## Discussion

Previous experiments using depletion approaches have identified a strong requirement in t(8;21) cells for the R/E oncoprotein for self-renewal, proliferation, and the block of differentiation (reviewed in Kellaway et al.^1^). However, which of these features were a direct consequence of R/E expression and which ones were only acquired during disease progression is unclear. Our study has shed light on the molecular details and the order of the earliest events during the transformation of myeloid progenitor cells by R/E, as schematically summarized in Figure 7E.

Once induced, R/E expression alters the GRN of myeloid progenitor cells and interferes with RUNX1 binding. The complete loss of RUNX1 binding leads to deregulation of cell cycle genes and a cell cycle block, the degree of which depends on whether R/E protein levels are in excess or equal to that of RUNX1. Our study provides a mechanistic explanation as to why balanced levels of wild-type and mutated RUNX1 are required in t(8;21) AML, which is also relevant to several other AML sub-types driven by translocations involving core binding factors.^52^^,^^53^^,^^54^ Moreover, our study also points to a wider requirement for RUNX1 expression in regulating growth and cell cycle progression, a function of importance to other types of AML such as the FLT3-ITD or *CEBPA* double mutant AML.^55^^,^^56^^,^^57^ It is clear that interference with RUNX1 activity in AML has immediate consequences with regards to the growth of early hematopoietic progenitor cells.

Dysregulation of cell proliferation is not the only effect of oncoprotein expression. R/E binding to regulatory elements linked to the *SPI1 (PU.1)* gene leads to the immediate down-regulation of this important myeloid TF and a block in differentiation at the CFU-GM progenitor stage. In addition, R/E activates an endothelial gene expression program and up-regulates MAP-kinase pathway genes, such as *JUN*, which in turn pushes myeloid progenitor cells toward an endothelial (arterial) identity. The acquisition of an endothelial gene expression signature was associated with the loss/absence of RUNX1 binding in both ESC and cord blood derived myeloid progenitor cells. Moreover, RUNX1 and R/E bound to the same sites in both up- and down-regulated genes specific to Art2 cells. The likely explanation for endothelial gene activation is the ability of RUNX1 to act as both activator and repressor of gene expression depending on the cellular context, with a major role in repressing endothelial genes.^17^ This finding provides a molecular explanation for the activation of endothelial genes seen in other RUNX1-related AMLs carrying the inv(16) or the t(3;21) translocations but also *CEBPA*-double mutant AMLs which share the feature of a block of myeloid differentiation.^54^^,^^57^^,^^58^

Another surprising result was the irreversibility of the reprogramming event that enforced an endothelial like cellular phenotype and the further progression of endothelial differentiation following R/E withdrawal. In mouse embryos, the re-activation of RUNX1 in a RUNX1 null background was sufficient to rescue the hematopoietic potential of the hemogenic endothelium, but only at early developmental stages, providing support for the idea that once endothelial differentiation has progressed, it is irreversible.^59^ R/E appears to halt this progress as well with cells sitting on the “edge” of endothelial and hematopoietic cell fates. This result is the likely explanation for the finding that RUNX1-ETO expression causes a disturbance in endothelial differentiation *in vitro*^19^ and in mice when expressed in the germline.^5^ Once R/E protein levels exceeded those of RUNX1 for extended periods of time as it was the case in 10 ng/mL Dox Matrigel cultures, reprogramming into endothelial cells was irreversible. RUNX1 protein was absent in these cultures, explaining why they were unable to down-regulate SOX17 and re-enter hematopoietic differentiation. However, t(8;21) patients did not show R/E mRNA in endothelial cells as measured by spatial transcriptomics (Merel van der Meulen and Olaf Heidenreich, Princess Maxima Center of Pediatric Oncology, personal communication), suggesting that even if formed in the embryo, such cells do not persist in adults.

An important result of our study with regards to the development of leukemic cells is the appearance of sub-populations of cells in the absence of exogenously added hematopoietic growth factors maintaining the hematopoietic gene expression program but only when RUNX1 and R/E levels are similar. In this cell population, differentiation is impaired but not completely blocked explaining why in t(8;21) patients the wild-type RUNX1 allele is never lost, and cells are still dependent on the presence of it for growth.^52^ However, the ability to grow was only evident during the co-culture of hematopoietic and reprogrammed endothelial cells and requires SCF, even after Dox withdrawal. The latter result suggests that normal SCF-expressing endothelial cells could serve the same function, a hypothesis that needs to be tested in future experiments.

We have previously shown in both cell lines and patient cells that depletion of R/E by siRNA-mediated knockdown promotes myeloid differentiation.^2^ As Dox was only withdrawn for 4 days, we have yet to test in the *in vitro* system whether R/E-exposed cells suffer from long-term effects of oncoprotein exposure with respect to their ability to differentiate. Indeed, a recent study showed that depletion of R/E in patient cells eliminated stemness features of leukemia initiating cells and differentiation from such cells was restricted to two lineages.^43^

Several recent studies have shown that RUNX1 function is intimately connected to growth factor signaling through its ability to cooperate with multiple signaling-responsive TFs, such as the mediators of MAP-kinase signaling, the AP-1 factor family. In t(8;21) patient blast cells, AP-1 is required to maintain leukemic growth^60^ and in t(8;21) LSCs, AP-1 mediates growth stimulation by VEGFA.^26^ In FLT3-ITD AML, RUNX1 chromatin binding is abolished in a subset of juxtaposed RUNX1-AP-1 binding sites when AP-1 binding is inhibited.^41^ Here, we show that the binding of R/E leads to an immediate up-regulation of MAP-kinase pathway genes suggesting that R/E-induced cells are ready to rewire their growth factor responsiveness. However, t(8;21) patient cells down-regulate such genes including those driving VEGF-mediated growth activation once the oncoprotein is depleted,^26^ demonstrating that maintaining RUNX1 vs. R/E balance is required throughout disease progression.

t(8;21) patients accumulate secondary mutations that facilitate the growth of R/E expressing cells, such as activating mutations in KIT. Our experiments shed light on how an endothelial environment could facilitate the acquisition of such mutations by maintaining a population of SCF responsive cells. Several studies, including those from our laboratory, have shown that introduction of an R/E expressing plasmid into human CD34^+^ HSPCs generates a population of growing myeloid progenitor cells whose proliferation can be further accelerated through overexpression of an activated KIT protein.^27^^,^^61^ In this setting, as in our system, R/E expression levels needed to be carefully calibrated to obtain proliferating cells. In addition, in such experiments, hematopoietic cells are grown in media supplemented with multiple cytokines, including SCF and may therefore select for cells that have acquired a GRN that can override the cell cycle block mediated by R/E in response to specific growth factors. Nevertheless, our data show that those cells still display the activation of an endothelial gene expression program, in particular activation of KDR, which could render a subset of such cells VEGF responsive. How the interplay between the different cytokines regulates the growth of preleukemic cells will be a major task in the future.

In summary, our study has demonstrated that ESC-derived HSPCs are a tractable system which enables dissection of the initial stages of cellular reprogramming of hematopoietic cells by oncogenic TFs. Our study also highlights the utility of such cells as a platform to identify molecular mechanism that may be targeted for future therapeutic interventions.

### Limitations of this study

Our *in vitro* experiments can only approximate what occurs when a hematopoietic precursor cell undergoes a chromosomal translocation. The exact cell type that initially is mutated *in vivo* is not known, nor are the soluble and cellular components of the niche that comprise the milieu where mutagenesis takes place. The production of hematopoietic growth factors such as SCF is also seen in normal endothelial cells.^49^ Presently, we do not know to which extent endothelial cells generated by R/E expression play a role different from those of normal endothelial cells at early stages of tumorigenesis. The Matrigel cultures demonstrate that signals from the arterial cells support hematopoietic cell growth but additional cells and signaling within the normal hypoxic bone marrow niche are likely to provide further support. That said, our study provides a clear explanation how the R/E-mediated cell cycle block can be bypassed.

## Resource availability

### Lead contact

Further information and requests for resources and reagents should be directed to and will be fulfilled by the lead contact, Andrew G. Elefanty (andrew.elefanty@mcri.edu.au).

### Materials availability

All cell lines can be shared and will be available from the lead contact upon request.

### Data and code availability

• ChIP and ATAC datasets from our previous publications were downloaded from the NCBI GEO database^19^ (GSE137673 and GSE137673, respectively, and GSE152335^27^). ScRNA-Seq data generated for this study were deposited at NCBI GEO (accession number GSE338360). Any additional information required to reanalyze the data reported in this paper is available from the lead contact upon request.
• Original new code has been deposited in https://doi.org/10.5281/zenodo.22218622.

## Acknowledgments

We thank S. Rowley (DNA Unit and Sequencing, Victorian Clinical Genetics Services) and colleagues for sequencing RNA samples, and M. Burton and E. Jones for flow cytometry assistance. This work was supported by the 10.13039/501100009708Novo Nordisk Foundation Center for Stem Cell Medicine, reNEW (10.13039/501100009708Novo Nordisk Foundation grant number NNF21CC0073729), the 10.13039/501100000925National Health and Medical Research Council of Australia (NHMRC) through research fellowships GNT1117596 (A.G.E.) and GNT1079004 (E.G.S.) and grants awarded to A.G.E. and E.G.S. (GNT1068866 and GNT1129861), to E.S.N. (GNT1164577 and GNT2012936), to A.G.E. (GNT2012535), and to E.G.S. (GNT1186019). We acknowledge funding by the Stafford Fox Medical Research Foundation, the Victorian government’s Operational Infrastructure Support Program, and the Australian government’s NHMRC Independent Research Institute Infrastructure Support Scheme. Previously published data from C.B.’s lab were funded by 10.13039/501100015570Blood Cancer UK (15001) and by a U21 studentship to A.G.E. and C.B.

## Author contributions

F.H., K.T., and L.F. performed experiments and analyzed data; A.M., P.K., and C.B. analyzed data; S.G.K. analyzed data and edited the paper; M.v.d.M. and O.T.H. provided unpublished data, conducted experiments, and edited the paper; E.G.S., E.S.N., A.G.E., and C.B. supervised the study; C.B. and A.G.E. wrote the paper. All authors approved the final version of the manuscript.

## Declaration of interests

A.G.E., E.S.N., and E.G.S. are consultants for Retro Biosciences Inc. The company was not involved in this study.

## Declaration of generative AI and AI-assisted technologies in the manuscript preparation process

During the preparation of this work, no AI-assisted technologies were used.

## STAR★Methods

### Key resources table

**Table:** 

REAGENT or RESOURCE	SOURCE	IDENTIFIER
**Antibodies**		

APC anti-human CD90	BD Biosciences	Cat# 559869 RRID:AB_398677
BV-421 anti-human CD34	BioLegend	Cat# 343610 RRID:AB_2561358
PeCy7 anti human CD45	BioLegend	Cat# 304016 RRID:AB_314404
APC anti-human CD144	BioLegend	Cat# 348508 RRID:AB_10639949
APC anti-human CXCR4	BioLegend	Cat# 306510 RRID:AB_314616
APC anti-human CD201	BioLegend	Cat# 351906 RRID:AB_11126139
BV-421 anti-human CXCR4	BioLegend	Cat# 306518 RRID:AB_11146018
BV-421 anti-human CD31	BioLegend	Cat# 303124 RRID:AB_2563810
BV-421 anti-human CD90	BioLegend	Cat# 328122 RRID:AB_2561420
PeCy7 antihuman CD309 (VEGFR2,KDR)	Biolegend	Cat# 393008 RRID:AB_2783297
APC anti-human Delta-like protein 4 (DLL4)	Biolegend	Cat# 346508 RRID:AB_11204071
APC anti-human CD117 (c-kit)	Biolegend	Cat# 313205 RRID:AB_314984
APC anti-human CD38	BioLegend	Cat# 303510 RRID:AB_314362
APC anti-human CD177	BioLegend	Cat# 313206 RRID:AB_314985

**Chemicals, peptides, and recombinant proteins**		

2-Mercaptoethanol	Sigma	Cat# M3148
Bone morphogenetic protein 4 (BMP4)	R&D Systems	Cat# 314-BP
Doxycycline	Stem Cell Technology	Cat# 72742
Dispase II	Thermo Fisher	Cat# 17105041
Epithelial growth fcator (EGF)	Peprotech	Cat# AF-100–15
Fibroblast growth factor 2 (FGF2)	Peprotech	Cat# 100-18B
FMS-like tyrosine kinase 3 receptor (FLT3) ligand	Peprotech	Cat# 300–19
Insulin-like growth factor 1 (IGF1)	Peprotech	Cat# 100–11
Insulin-like growth factor 2 (IGF2)	Peprotech	Cat# 100–12
Interleukin 3 (IL3)	Peprotech	Cat# 200–03
Interleukin 6 (IL6)	Peprotech	Cat# 200–06
Matrigel	Corning	Cat# 356231
Penicillin/Streptomycin (Pen/Strep)	Thermo Fisher	Cat# 15140122
SPELS medium	Ng et al.^31^	Table S23
Stem cell factor (SCF)	Peprotech	Cat# 300–07
Thrombopoietin (TPO)	Peprotech	Cat# 300–18
ViaStain Acridine Orang/Propidium iodide (AOPI) staining solution	Revvity	Cat# CS2-0105
Vascular endothelial growth factor (VEGF)	Peprotech	Cat# 100–20
Ripretinib (DCC-2618) KIT inhibitor	Selleck Chemicals	Cat# S8757

**Critical commercial assays**		

Chromium fixed RNA kit human transcriptome	Millennium Science	Cat# 10x-1000475
Chromium next gem chip Q	Millennium Science	Cat# 10x-1000422
Chromium next gem single cell fixed RNA sample PREP kit	Millennium Science	Cat# 10x-1000414
Isolate II RNA Micro Kit	Meridian Bioscience	Cat# BIO- 52075
Isolate II RNA Mini Kit	Meridian Bioscience	Cat# BIO-52073
Tetro cDNA synthesis kit	Meridian Bioscience	Cat# BIO-65043

**Deposited data**		

scRNA-seq data	This study	NCBI GEO: GSE338360

**Experimental models: Cell lines**		

Human: Inducible RUNX1-ETO SOX17^mCherry/w^RUNX1C^GFP/w^ hESC H9 line	Nafria et al.^18^	N/A

**Oligonucleotides**		

*CD34*	Thermo Fisher Scientific	RRID: Hs00156373_m1
*CXCR4*	Thermo Fisher Scientific	RRID: Hs00237052_m1
*DLL4*	Thermo Fisher Scientific	RRID: Hs00184092_m1
*GAPDH* (TaqMan assay)	Thermo Fisher Scientific	RRID: Hs99999905_m1
*HLF* (TaqMan Assay)	Thermo Fisher Scientific	RRID: Hs00171406_m1
*KDR* (TaqMan Assay)	Thermo Fisher Scientific	RRID: Hs00176676_m1
*MECOM* (TaqMan Assay)	Thermo Fisher Scientific	RRID: Hs01118143_m1
*PECAM1* (*CD31)* (TaqMan Assay)	Thermo Fisher Scientific	RRID: Hs00169777_m1
*RUNX1 Common* (TaqMan Assay)	Thermo Fisher Scientific	RRID: Hs00231079_m1
*RUNX1C* (TaqMan Assay)	Thermo Fisher Scientific	RRID: Hs01021967_m1
*RUNX1-ETO* (TaqMan Assay)	Thermo Fisher Scientific	GTACGATTTCGAGGTTCTCGGGG
*SPI1 (PU.1)* (TaqMan Assay)	Thermo Fisher Scientific	RRID: Hs00231368_m1
*TEK (TIE2)* (TaqMan Assay)	Thermo Fisher Scientific	RRID: Hs00176096_m1

**Software and algorithms**		

Single cell RNA-Seq analyses	This study	https://doi.org/10.5281/zenodo.22218622
Gene regulatory network analysis	Cite https://doi.org/10.1016/j.celrep.2023.113568	https://github.com/petebio/Gene_regulatory_network_analysis
ChIP data re-analysis	This study	https://doi.org/10.5281/zenodo.22218622
Cell Ranger (v7.2.0)	https://www.10xgenomics.com/	https://support.10xgenomics.com/single-cell-gene-expression/software/downloads/latest
Seurat (v5.1)	Cite https://doi.org/10.1038/s41587-023-01767-y	https://satijalab.org/seurat/
Monocle3 (v1.3.26)	Cite https://doi.org/10.1038/nmeth.4402	https://cole-trapnell-lab.github.io/monocle3/
Slingshot (v2.16.0)	Cite https://doi.org/10.1186/s12864-018-4772-0	https://github.com/kstreet13/slingshot
tradeSeq (v1.22.0)	Cite https://doi.org/10.1038/s41467-020-14766-3	https://github.com/statOmics/tradeSeq/
Trimmomatic (v0.39)	Cite https://doi.org/10.1093/bioinformatics/btu170	https://www.usadellab.org/cms/?page=trimmomatic
Bowtie2 (v2.5.4)	Cite https://doi.org/10.1038/nmeth.1923	https://bowtie-bio.sourceforge.net/index.shtml
Picard	https://broadinstitute.github.io/picard	N/A
MACS2 (v2.2.9.1)	Cite https://doi.org/10.1186/gb-2008-9-9-r137	https://github.com/macs3-project/MACS
HOMER (v4.11)	Cite https://doi.org/10.1016/j.molcel.2010.05.004	https://homer.ucsd.edu/homer/
R (v4.4.1)	https://www.r-project.org/	N/A
enrichR (v3.4)	Citation https://doi.org/10.32614/CRAN.package.enrichR	https://cran.r-project.org/web/packages/enrichR/index.html
Java TreeView (v3.0)	Cite https://doi.org/10.1093/bioinformatics/bth349	https://jtreeview.sourceforge.net/
Python v3.12.3	https://www.python.org/	N/A
Pybedtools v0.11.0	Cite https://doi.org/10.1093/bioinformatics/btr539	https://daler.github.io/pybedtools/
Bedtools (v2.31.1)	Cite https://doi.org/10.1093/bioinformatics/btq033	https://bedtools.readthedocs.io/en/stable/
Subread (v2.0.6)	Cite https://doi.org/10.1093/bioinformatics/btt656	https://subread.sourceforge.net/
GraphPad Prism	https://www.graphpad.com/	N/A

**Plastics**		

6-well low attachment plates	Corning	Cat# 3516
24-well low attachment plates	Corning	Cat# 3524

### Experimental model and study participant details

The RUNX1-ETO expressing human ESC line used in this study has been described in.^19^

### Method details

#### RUNX1-ETO induction experiments

Hematopoietic stem and progenitor cells (termed iHSCs) were generated as described^31^ and harvested from days 13–15 and cryopreserved in CJ2/10% DMSO cryopreservation medium.^62^ iHSCs were thawed and cultured in SPELS medium base^31^ supplemented with 100ng/mL recombinant human (rh) stem cell fator (SCF), 100ng/mL rh thrombopoietin (TPO), 25 ng/mL rh interleukin (IL)-3 and 2U/mL rh erythropoietin (EPO). Cells were cultured on an orbital rotating platform in 6-well plates for up to 8 days and RUNX1-ETO was induced using between 0 and 10ng/mL of doxycycline (Dox). Culture medium, including dox, was replenished every 2 days, and cell samples were taken for growth analysis, flow cytometry analysis and mRNA expression analysis. For growth analysis, cells were harvested every two days and counted using an automated cell counter (Nexcelcom Biosciences) using Propidium Iodide/Acridine Orange staining for live/dead cell discrimination. Cells were analyzed by flow cytometry (BD Fortessa).

#### Western blots

D17 iHSCs were expanded in myeloid expansion medium for four days in the presence of no dox, 5ng/mL dox or 10ng/mL of dox. For total cell extracts cells were harvested and lysed using RIPA buffer (Thermo Fisher, Cat#89900). Proteins were separated using NuPage 4–12% Bis-Tris Gels (Thermo Fisher Cat#26616) and PageRuler (Thermo Fisher Cat#26616) was used as a molecular weight marker. Proteins were transferred onto a nitrocellulose membrane using the *Trans*-blot turbo blot transfer system (Biorad, Cat# 1704150EDU). Non-specific protein binding was blocked by 1-h incubation in 5% Milk in PBS/Tween (PBS +0.1% Tween 20) followed by primary antibody staining using AML1 antibody (Cell Signaling, Cat#4334). Following an overnight incubation in primary antibody, cells were washed 3 times in PBS/Tween and then incubated for 1 h in anti-GAPDH (Abcam, Cat#8245) secondary antibody. Nuclear and cytoplasmic protein extractions were prepared using the Ner-Per nuclear and cytoplasmic protein extraction protocol and reagents (Thermo Fisher, Cat#78833). Protein separation and transfer following nuclear and cytoplasmic extraction was performed as described previously. Proteins were detected using SignalFire ECL Reagents (Cell Signaling, Cat#6883P3) and imaged using Amersham Imager 680.

#### RUNX1-ETO withdrawal experiments and matrigel assay

Day 15 iHSCs were cultured for 6 days in myeloid expansion medium and treated with either 0, 5 or 10ng/mL of dox, replenished every 2 days. On day 2, dox treated cells were flow cytometrically sorted for CD90^+^CD34^+^ expression. Since only induced cells expressed CD90, uninduced cells were sorted based on CD34^+^ expression.

After 6 days of myeloid expansion, cells were replated onto Matrigel coated 6 well plates where dox treatment was either continued or withdrawn for a further 2 days. Cells were maintained in SPELS base medium supplemented with 2ng/mL rh bone morphogenetic protein 4 (BMP4), 5ng/mL rh insulin-like growth factor (IGF)1 and rh IGF2, 10ng/mL rh fibroblast growth factor (FGF) 2, 5ng/mL rh epidermal growth factor (EGF) and 25ng/mL rh vascular endothelial growth factor (VEGF). Cells were harvested at day 8 or day 10 (2 or 4 days post plating into Matrigel) by gently aspirating floating cells followed by rinsing the well with SPELS. 1mL of 2ng/mL Dispase was added to each well and incubated for approximately 15 min at 37°C on a rotating platform at 60rpm. The wells were then flushed using SPELS to lift adherent cells. Harvested cells were analyzed using flow cytometry and qPCR. To examine the role of SCF in co-cultures we added 64 nm of the KIT inhibitor Ripretinib (see below). To examine cultures without endothelial cells, hematopoietic cells growing in suspension were replated in the same medium and cultured in the absence of endothelial cells for 2 more days.

#### Cytokine culture and inhibition experiments

After 6 days of myeloid expansion, cells were maintained in SPELS basal medium supplemented with 2 ng/mL recombinant human (rh) bone morphogenetic protein 4 (BMP4), 5 ng/mL rh insulin-like growth factor 1 (IGF1), 5 ng/mL rh insulin-like growth factor 2 (IGF2), 10 ng/mL rh fibroblast growth factor 2 (FGF2), and 5 ng/mL rh epidermal growth factor (EGF). Cultures were maintained in 6-well plates on an orbital rotating platform for 4 days. Cells were cultured with or without 25 ng/mL recombinant human vascular endothelial growth factor (VEGF), 100 ng/mL recombinant human stem cell factor (SCF), or both factors, and in the presence or absence of 64 nM of the KIT inhibitor Ripretinib, which was the minimal effective concentration as previously determined by the same assay. Doxycycline (5ng/mL) was replenished every 2 days throughout the culture period. Harvested cells on days 2 and 4 were counted and analyzed by flow cytometry.

#### Cell sorting and flow cytometry

Cell surface marker expression of hematopoietic cells was analyzed by flow cytometry using a BD LSRFortessa flow cytometer. Briefly, cell pellets were stained with antibodies for 15 min in the dark and washed with FACS wash buffer before resuspending in wash buffer containing propidium iodide to identify viable cells.

Hematopoietic cells were also single cell sorted for CD90 and CD34 cell surface expression using the BD FACSAria. A complete list of antibodies used for flow cytometry can be found in the STAR Methods table.

#### Methylcellulose colony-forming assays

Hematopoietic colony forming cells in RUNX1-ETO induced and uninduced cultures were identified by culturing 500 cells in serum free methylcellulose, supplemented with 100ng/mL rh TPO, 100ng/mL rh SCF, 25ng/mL rh IL3, 25ng/mL rh IL6, 25ng/mL rh FLT3-ligand (FLT3L), 25ng/mL rh granulocyte colony stimulating factor (G-CSF) and 5U/mL rh EPO. Methylcellulose medium was prepared by mixing 40 mL of serum-free MethoCult™ H4100 with an equal volume of IMDM 2*X* supplement. The IMDM 2*X* supplement contained IMDM with 2*x* ITS-E, 0.2% rh albumin (Sartorius), 0.03% monothioglycerol (MTG), 0.2*x* SyntheChol, 125 ng/mL linolenic and linoleic acids, 1 μg/mL soybean oil, 2 mM GlutaMAX-I, 50 μg/mL L-ascorbic acid 2-phosphate (AA2P), and 50 μg/mL ascorbic acid. To this mixture, 20 mL of SPELS medium was added to achieve a final volume of 100 mL, along with 5 μM hydrocortisone and 9 ng/mL human low-density lipoprotein (hLDL). Cells were cultured in methylcellulose in the presence or absence of 5 or 10 ng/mL Dox. Dox was added to the methylcellulose every two days. Cultures were set up in duplicate in low attachment 24-well plates. Plates were scored for total hematopoietic colonies after 6 days and for colony subtypes after 16 days.

#### Confocal images

D15 iHSCs were cultured in myeloid expansion medium with either 0 dox, 5ng/mL dox or 10ng/mL of dox for 6 days. On day 6, 100k cells were seeded onto 24 well plates, precoated overnight with 200ul of neat Matrigel. Wells were topped up with 200ul of media supplemented with 25ng/mL BMP4, 5ng/mL IGF1, 5ng/mL IGF2, 5ng/mL EGF, 10ng/mL bFGF, 25ng/mL VEGF and no dox, 5 dox, 10 dox or dox withheld, respectively.

Images were taken 48hours post plating using a Zeiss LSM900 confocal microscope. The red color indicates the expression of the SOX17 mCHERRY reporter.

#### RNA extraction and real time quantitative PCR analysis

Total RNA was extracted from cell samples using the Isolate II RNA Micro Kit (Bioline) following the manufacturer’s instructions. Complementary DNA (cDNA) was synthesized from purified RNA using random hexamer priming and the the Bioline Tetro cDNA Synthesis Kit (Bioline). Quantitative PCR (qPCR) was performed using cDNA as a template in combination with TaqMan Fast Advanced Master Mix (Thermo Fisher) and the TaqMan Probes described in the key resources table in a MicroAmp™ 96-well optical reaction plate. *GAPDH* (Hs99999905_m1) expression was used as a reference control. Reactions were run on a QuantStudio™ 5 Flex Real-Time PCR System (Applied Biosystems).

### Quantification and statistical analysis

Statistical analysis methods used in bioinformatic analysis are listed in the bioinformatic methods. For other figures, the relevant methods are stated in the figure legends. Exact numbers of biological and technical replicates for each experiment are reported in the figure legends. *p* values less than 0.05 were considered statistically significant by unpaired, two-tailed Student’s *t* test and are indicated in the relevant figures.

#### Single-cell RNA sequencing

##### Sample generation

Single-cell suspensions were generated from the different cultures and cells were fixed using the 10*x* Genomics Fixation Kit for Chromium Fixed RNA Profiling (CG000478 Rev C) according to the manufacturer’s instructions. Cell viability was assessed using a Nexcelcom Biosciences cell counter with acridine orange/propidium iodide (AOPI) staining. All samples were fixed overnight for 24 h at 4°C and subsequently stored at −80°C until library preparation.

Samples were barcoded using the multiplexed human transcriptome kit for fixed RNA (10*x* Genomics PN-1000476), allowing sequencing of four samples in a single GEM reaction. Libraries were prepared using the 10*x* Genomics Chromium Controller, targeting 6,000 cells per sample. Barcoded single-cell cDNA libraries were generated and amplified according to the manufacturer’s workflow. Libraries were sequenced on an Illumina NovaSeq 6000 platform with paired-end reads to a depth of approximately 50,000 reads per cell.

##### Data processing and clustering

Raw base call files were converted to FASTQ format and aligned to the GRCh38–1.2.0 reference genome using Cell Ranger (v7.2.0) with default parameters. Cell barcodes and unique molecular identifiers (UMIs) were filtered and counted to generate gene expression matrices.

Downstream analyses were performed in R (v4.4.1) using Seurat (v5.1). Cells with fewer than 2,500 detected genes or more than 5% mitochondrial transcripts or more than 1x10^5^ transcripts were excluded. Gene expression matrices were log-normalized. Dimensionality reduction was performed using principal component analysis (PCA) followed by UMAP for visualization. Clustering was conducted using a shared nearest neighbor graph-based approach with resolution set to 0.3. Following initial clustering only those cluster containing more than 100 cells were included in downstream analyses.

Differential gene expression was assessed using the FindMarkers function of Seurat. Cell types were annotated based on canonical marker gene expression and confirmed by reference-based annotation using scType and comparison to known markers of HSPC subtypes as shown in.^31^^,^^32^

##### Single-cell trajectory analysis

The processed single-cell expression data object from Seurat was imported into Monocle3 v1.4.26^63^ using the as.cell_data_set function from SeuratWrappers (https://github.com/satijalab/seurat-wrappers). Trajectories were then inferred using the learn_graph command in Monocle with the parameters close_loop = FALSE and use_partition = FALSE. Pseudotime was then calculated using the order_cells function using earliest principal node in the HSC population as the root node. These were then plotted using the UMAP coordinates calculated by Seurat.

#### Classification of RUNX1 and RUNX1-ETO binding patterns from ChIP-Seq data

ChIP-Seq data were down-loaded from GSE137673 and re-processed. Peak sets representing distinct RUNX1 and RUNX1-ETO binding patterns were defined using a custom python script (see code availability section). To do this, BED files from ChIP-Seq experiments were first filtered to keep only peaks found in open chromatin by comparing them to the union of all ATAC-Seq peaks using the intersect function in the pybedtools package v0.11.0^64^ in python v3.12.3. Only peaks that were found within an ATAC peak were kept for further analysis.

To ensure consistency in peak annotations between different ChIP-Seq experiments, peaks were mapped to a common set of coordinates by combining peaks from all experiments into a single pybedtools data object and merging them using the merge function. ChIP binding patterns were then classified by comparing the peaks from the RUNX1 0 dox, RUNX1 5 dox and RUNX1-ETO 5 dox samples to each other using the intersect function in pybedtools. The set of queries used to do this can be found in the *define_peak_sets.py* script (see code availability section). The resultant peak sets were then exported as BED files.

ChIP peaks were annotated to their target genes using *annotateBED_with_CHiC.py* script from Coleman et al. (2023).^55^ This was done by comparing ChIP peaks in distal *cis*-regulatory elements to sites annotated to the promoter that they interact with using promoter-capture HiC datasets obtained from healthy PBSCs and t(8;21) AML patients. In cases where a peak could not be annotated with the HiC data, or was found at a promoter site, peaks were instead annotated to their closest gene using the annotatePeaks.pl function in HOMER v4.11.^65^ The *annotateBED_with_CHiC.py* script and promoter-capture HiC annotation files used for this analysis can be found in the GitHub repository for Coleman et al. (2023)^55^ at https://github.com/petebio/Gene_regulatory_network_analysis.

##### Comparison of ChIP-Seq binding patterns to changes in gene expression along the HSC to Art2 differentiation trajectory

Genes that change expression during Art2 differentiation after RUNX1-ETO induction were identified by selecting the single-cell clusters corresponding to earliest HSCs in the 0 dox sample and comparing these to the Art2 cells at the terminal end of the differentiation trajectory in either the 5 or 10 dox samples. This was done using the FindMarkers function in Seurat with the option min.pct = 0.1 to keep only genes that were detected in at least 10% of cells. A gene was considered to have significantly changed expression if it had a fold-change greater than 2 and a Bonferroni adjusted *p*-value less than 0.01.

To test if the set of up or down-regulated genes could be associated with specific changes in the pattern of RUNX1 and/or RUNX1-ETO binding, we compared them to the ChIP target gene sets associated with specific binding patterns. This was done using a one-sided (right-tailed) Fisher’s exact test in R v4.5.0 with the resulting *p*-values adjusted for multiple testing using the Benjamini-Hochberg method. Any comparison that had an adjusted *p*-value less than 0.05 was considered statistically significant.

KEGG pathway analysis was then conducted on the up or down-regulated ChIP target genes from significant comparisons using the enrichR package v3.4^66^ in R and using all genes that were expressed in at least 10% of cells in the single-cell clusters as the background gene set. Pathways with a Benjamini-Hochberg adjusted *p*-value less than 0.05 was considered statistically significant.

##### Analysis of gene expression along trajectories

Slingshot (version 2.16.0)^67^ was used to infer trajectories on UMAP embeddings. Cluster 1 was set as the starting population and the clusters 5, 3, 4 and 6 were specified as end clusters. Slingshot was run with the commands extend = “n”, allow.breaks = TRUE, reweight = True, thresh = 0.01 and maxit = 30.

tradeSeq (version 1.22.0)^34^ was then used to fit a negative-binomial generalised additive model for each gene along pseudotimes. 1500 cells sampled randomly were selected to be used in the model split equally across the lineages. The resulting set of cells were used to construct pseudotime matrices, lineage weight matrices and count matrices for tradeSeq analysis. Models were fit using nknots = 9. associationTest was used to identify genes with differential expression along pseudotime.

For lineage specific pseudotime clustering, cells belonging to slingshot Lineage 1 (10 dox) and Lineage 2 (5 dox) were selected. Genes with less than 20 counts across the lineage were removed. Differentially expressed genes along the lineages were identified using tradeSeq and then filtered to include genes with padj<0.05 and which were in the top 40% ranked by absolute log fold-change. Smoothed expression trajectories were estimated across pseudotime for each gene using predictSmooth function. Gene by pseudotime matrices were then scaled by *Z* score scaled per gene.

To determine the number of clusters to use for K-means clustering,^68^ K-means was run for k = 1–15 and the total within-clusters sum of squares was plotted to form an elbow plot. Final gene clusters were generated using R’s kmeans() function with centers = 4 and nstart = 25.

##### ATAC-seq data processing

Single-end ATAC-seq reads from sorted RUNX1c-positive and RUNX1c-negative populations were down-loaded from GSE137673 and were reprocessed by trimming using Trimmomatic (v0.39)^69^ to remove low-quality bases and Nextera adaptors. Reads were aligned to the human genome (hg38) using Bowtie2 (v2.5.4)^70^ with the --very-sensitive-local parameter. PCR duplicates were removed with Picard MarkDuplicates (v2.26.10). Peaks were called using MACS2 (v2.2.9.1)^71^ with the parameters --nomodel -B --trackline and filtered against the hg38 ENCODE blacklist^72^ using bedtools (v2.31.1).^73^ Peaks were extended ±200 bp around the summit and annotated with HOMER annotatePeaks.pl.^65^

##### ChIP-seq data processing

ChIP-seq reads were trimmed, aligned, and filtered as described above. Peaks were called with MACS2 using -B --tracklineand extended ±100 bp around the summit. ChIP-seq peaks were filtered against the merged ATAC-seq peaks from positive and negative RUNX1c populations to remove sites never accessible in ATAC-seq. Filtered peaks were annotated using the published annotate_bed_with_HiC pipeline,^55^ which integrates CHiC interaction data with HOMER’s annotatePeaks.pl to assign peaks to target genes.

##### Regulatory element classification

Annotated peaks were intersected across Dox0 and Dox5 RUNX1 samples and RUNX-ETO Dox5 samples using Intervene Venn^74^ to define seven groups of regulatory elements as marked in Figure 2A. These groups formed the basis for integration with scRNA-seq trajectories.

##### Integration of regulatory elements with scRNA-seq trajectory

To assess the functional consequences of RUNX1 and RUNX-ETO binding on gene expression, the regulatory element groups were integrated with scRNA-seq pseudotime trajectories. Cells at the extremes of the pseudotime trajectory were selected to ensure accurate fold-change estimates and to minimize confounding from intermediate heterogeneous populations.

Differential gene expression between trajectory extremes was calculated using Seurat’s **FindMarkers** function, and genes were assigned to the seven groups based on prior ChIP-seq annotation. Gene set enrichment (GSEA,^75^) and Leading-edge analyses were performed for each group to identify pathways and genes associated with distinct transcription factor binding patterns.

#### Reanalysis of CD34^+^ cord blood and primary AML ATAC-seq and ChIP-seq data

ATAC-seq and ChIP-seq fastq files were downloaded from GSE152335 and GSE34540, trimmed, aligned and subject to peak-calling as above. Accessible chromatin sites corresponding to Art2-specific genes (>2-fold differential vs. HSPCs) were identified in the CD34^+^ cord blood ATAC-seq using a custom python script (see code deposition) based on assigning the nearest gene to each open chromatin peak. ATAC and ChIP-seq reads were then counted for each site using featureCounts from the Subread package v2.0.6^76^ using the accessible site coordinates as the input SAF file and the option -F SAF. Counts were hierarchically clustered in R version 4.5.3 using complete linkage and plotted as a heatmap. Row order following clustering was used to extract genes to plot Art2 vs. HSPC differential expression.

Separately, a peak union was generated from RUNX1c-positive ATAC-seq peaks (see above), and the average tag-density was calculated for each sample at these peaks using the annotatePeaks.pl function in Homer v4.11 using the bedGraph files generated by MACS2. Tag densities were then normalised as CPM, a pseudocount of 0.1 was added and log_2_ 10Dox10/0Dox fold change was calculated. Peaks were ranked from high to low fold change and this ranking was used for subsequent density plots. Density plots were generated using Homer v4.11 annotatePeaks.pl function using the bedGraph files generated by MACS2, with the options -size 2000 -hist 10 -ghist and plotted using JavaTreeView 1.1.6.

#### Differential gene expression analysis of arterial cell types

Differential gene expression analysis between continuous and discontinued doxycycline-treated arterial cell populations was performed using the FindMarkers function in Seurat. Genes were tested with Wilcoxon rank-sum test implemented in Seurat. Genes were required to be detected in at least 10% of cell to in either comparison. Differentially expressed genes were ranked by average log2 fold-change and adjusted *p*-value. Genes were considered differentially expressed if they had an absolute average log2 fold-change of at least 0.25 and an adjusted *p*-value below 0.05. Volcano plots were generated using EnhancedVolcano package in R. Gene ontology enrichment analysis was performed on differentially expressed genes between the two conditions. Genes which were 2 fold-change upregulated with an adjusted *p* value threshold 0.05 were taken for gene ontology analysis using the R package clusterProfiler^77^ using the human genome annotation database org.Hs.e.g.,db.

## Supplemental information

Document S1. Figures S1–S7

Data S1. Single-cell RNA-Seq analysis of uninduced and Dox-induced HSPCs Gene expression values in Seurat clusters (related to Figure 1E). Avg_Log2FC: average log2 fold change in expression.

Data S2. Slingshot analysis of RNA-Seq data from uninduced and Dox-induced HSPCs *k*-means clustering of data from 5 Dox-induced cells (related to Figure S2D).

Data S3. Slingshot analysis of RNA-Seq data from uninduced and Dox-induced HSPCs *k*-means clustering of data from 10 Dox-induced cells (related to Figure S2E).

Data S4. Differential gene expression (DEG) between HSPCs and Seurat Art2 clusters for 5 Dox-induced cells (Tab A) and 10 Dox-induced cells (Tab B) Avg_Log2FC: average log2 fold change in expression. Related to Figures 3C and S3C, respectively

Data S5. Underlying data analyses for Kegg pathway definition (related to Figures 3 and S3). Tab A: 5 Dox-induced cells, Tab B: 10 Dox-induced cells, Tab C: full list of genes in enriched KEGG pathway associated with gained and up-regulated RUNX1-ETO peaks; Tab D: full list of genes in enriched KEGG pathway associated with lost and down-regulated RUNX1 peaks

Data S6. Single-cell RNA-Seq analysis of Dox-induced and de-induced HSPCs Avg_Log2FC: average log2 fold change in expression. Gene expression values in Seurat clusters (Tabs 0–13 merging day 14 and day 22 induced cells. Related to Figure S6B.

Data S7. Single-cell RNA-Seq analysis uninduced HSPCs Gene expression values in Seurat clusters (Tabs 0–11) in uninduced (0 Dox) cells cultured for 14 and 22 days (related to Figure 6C).

Data S8. Single-cell RNA-Seq analysis of Dox-induced and de-induced HSPCs Avg_Log2FC: average log2 fold change in expression. Gene expression values in Seurat clusters of day 22 induced and de-induced cells (Tabs 0–11). Related to Figure 6C.

Data S9. Gene expression values for the genes in the arterial clusters 7 and 9 before and after Dox withdrawal Avg_Log2FC: average log2 fold change in expression (related to Figures 6C and 7A).

Data S10. After RUNX1-ETO withdrawal 5 Dox-induced Art2 cells resume endothelial differentiation Tab 1, differential gene expression analysis of genes from 5 Dox-induced and de-induced cells. Avg_Log2FC: average log2 fold change in expression. Tab 2, genes up-regulated in the discontinued cluster 7. Tab 3, GO-terms enriched in cluster 7 + 9; Tab 4: GO terms enriched in the discontinued cluster 7. Related to Figures S7D and S7E.

Document S2. Article plus supplemental information
