# Supplementary material for: RUNX1-ETO oncoprotein levels determine the balance between endothelial reprogramming and pre-leukemic growth in a human ES cell model of AML
Source: Cell Rep. 2026 Oct 19;45(10):118157. doi: 10.1016/j.celrep.2026.118157 (PMC13714367; doi:10.1016/j.celrep.2026.118157)
Supplement: Supplementary file 1 [file mmc1.pdf]

Cell Reports, Volume *45*

Supplemental information

RUNX1-ETO oncoprotein levels determine the balance between endothelial reprogramming and pre-leukemic growth in a human ES cell model of AML

Katerina Terolli, Floyd Hassenrueck, Alexander Maytum, Peter Keane, Lea Flippe, Sophie G. Kellaway, Merel van der Meulen, Olaf T. Heidenreich, Edouard G. Stanley, Elizabeth S. Ng, Andrew G. Elefanty, and Constanze Bonifer

SUPPLEMENTARY FIGURES AND FIGURE LEGENDS

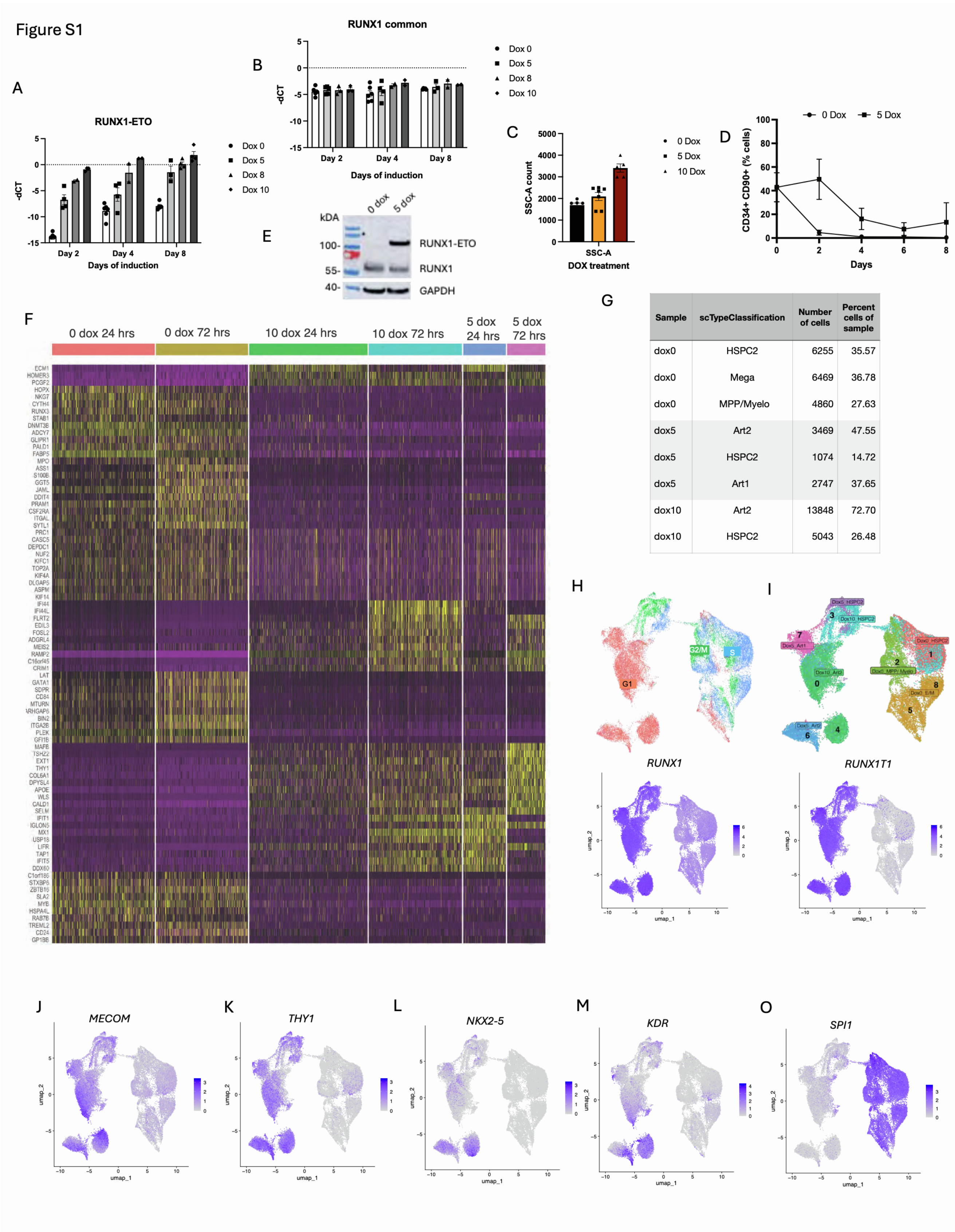

Sample	scTypeClassification	Number of cells	Percent cells of sample
dox0	HSPC2	6255	35.57
dox0	Mega	6469	36.78
dox0	MPP/Myelo	4860	27.63
dox5	Art2	3469	47.55
dox5	HSPC2	1074	14.72
dox5	Art1	2747	37.65
dox10	Art2	13848	72.70
dox10	HSPC2	5043	26.48

Supplementary Figure 1 (related to Figure 1)

Expression of RUNX1-ETO leads to dosage-dependent arterial patterning and a cell cycle block

A, B. Real-Time PCR analysis using Taqman probes showing transcript levels of RUNX1-ETO (A) and RUNX1 (B) from d2-d8 after induction with different doxycycline concentrations (0,5,8,10 ng/mL). Error bars represent s.e.m. for n=3

C: Increase in cell size as measured by SSC after 5 and 10 Dox induction as compared to the control (0 Dox). Error bars represent s.e.m. for n=4

D. Percentage of CD34+ and CD90+ cells during 8 days of induction with 5 Dox as compared to the control (0 Dox)

E. Western blot of total cell extract of HPSCs following four days of expansion in the presence of no dox, 5ng/ml or 10ng/ml of dox. Anti- AML1 (55kDA) antibody was used to detect RUNX-ETO (100kDA) fusion protein and endogenous RUNX1 (55kDA) protein levels. GAPDH (36 kDa) was used as a loading control.

F. Heatmap of cluster representative genes used to determine sample identity based on human embryo data as in reference [1]. The purple colour represents low, and the yellow colour represents high level expression compared to all other samples.

G Number of cells in each cluster of Fig. 1 E.

H. UMAP representation of the cell cycle status in the different Seurat clusters depicted in **I**

i. Upper right panel: Cell type classification of induced and uninduced cells as in Fig 1 E. Left bottom panel: Feature plot depicting the expression of RUNX1 in each cluster. Bottom right panel: Expression of RUNX1T1 in each cluster. Note that in contrast to the Taqman probes, the scRNA-Seq probes cannot distinguish between RUNX1T1 and RUNX1-ETO.

J.- O.: Feature plots depicting the expression levels of the indicated genes in the different Seurat clusters.

Figure S2

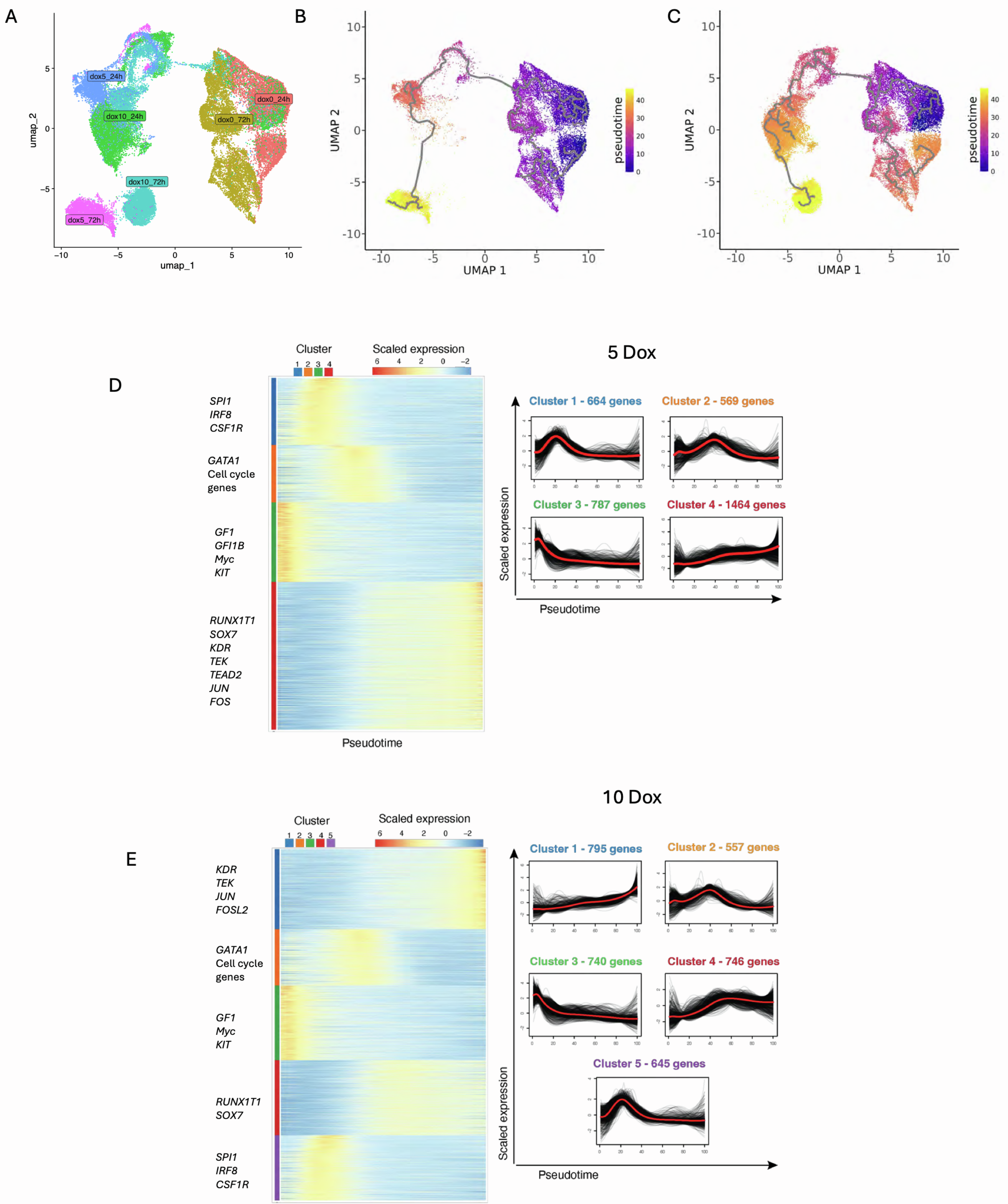

A
B
C
D
E
dox5_24h
dox10_24h
dox0_24h
dox0_72h
dox10_72h
dox5_72h
umap_1
umap_2
UMAP 1
UMAP 2
pseudotime
5 Dox
10 Dox
Cluster
Scaled expression
SPI1
IRF8
CSF1R
GATA1
Cell cycle genes
GF1
GFI1B
Myc
KIT
RUNX1T1
SOX7
KDR
TEK
TEAD2
JUN
FOS
FOSL2
Pseudotime
Cluster 1 - 664 genes
Cluster 2 - 569 genes
Cluster 3 - 787 genes
Cluster 4 - 1464 genes
Cluster 1 - 795 genes
Cluster 2 - 557 genes
Cluster 3 - 740 genes
Cluster 4 - 746 genes
Cluster 5 - 645 genes

Supplementary Figure 2 (related to Figure 2)

Expression of RUNX1-ETO alters the developmental control of gene expression and the differentiation trajectory

A: Annotation of Seurat clusters according to the time and concentration of Dox induction as indicated.

B – C: Pseudo-time analysis of the developmental trajectory of cells without and after 5 ng/ml (B) and with and without 10 ng/ml Dox (C) using Monocle.

D: K-means clustering of the developmental trajectories of co-regulated genes with and without 5 ng/ml RUNX1-ETO induction from tradeSeq analysis. Examples of such genes are plotted on the left of the heat map.

E: K-means clustering of the developmental trajectories of co-regulated genes with and without 10 ng/ml RUNX1-ETO induction from tradeSeq analysis. Examples of such genes are plotted on the left of the heat map.

Figure S3

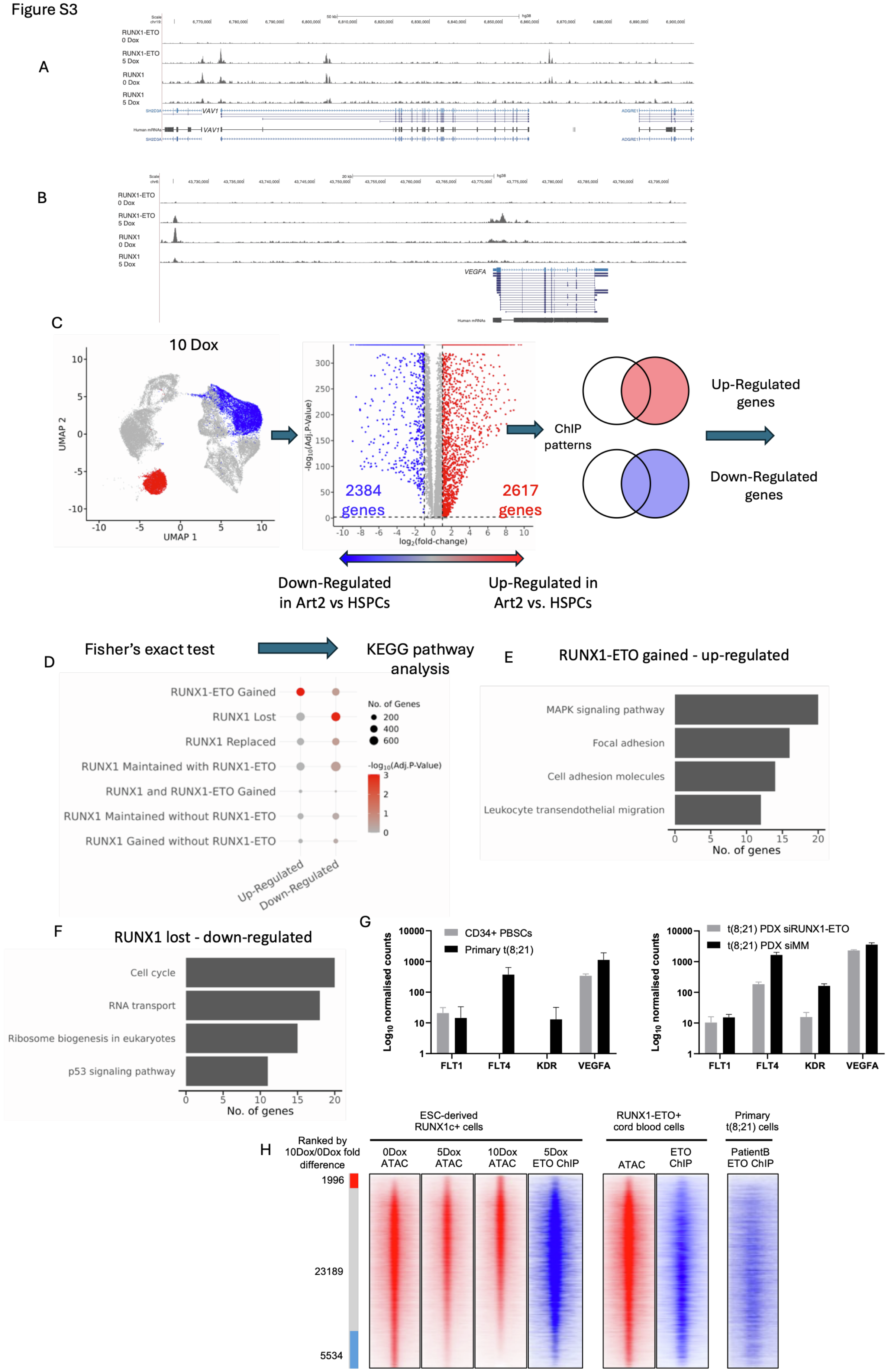
A
RUNX1-ETO 0 Dox
RUNX1-ETO 5 Dox
RUNX1 0 Dox
RUNX1 5 Dox
VAV1
B
RUNX1-ETO 0 Dox
RUNX1-ETO 5 Dox
RUNX1 0 Dox
RUNX1 5 Dox
VEGFA
C
10 Dox
UMAP 2
UMAP 1
-log10(Adj.P-Value)
log2(fold-change)
2384 genes
2617 genes
ChIP patterns
Up-Regulated genes
Down-Regulated genes
Down-Regulated in Art2 vs HSPCs
Up-Regulated in Art2 vs. HSPCs
Fisher's exact test
KEGG pathway analysis
D
RUNX1-ETO Gained
RUNX1 Lost
RUNX1 Replaced
RUNX1 Maintained with RUNX1-ETO
RUNX1 and RUNX1-ETO Gained
RUNX1 Maintained without RUNX1-ETO
RUNX1 Gained without RUNX1-ETO
Up-Regulated
Down-Regulated
No. of Genes
200
400
600
-log10(Adj.P-Value)
E
RUNX1-ETO gained - up-regulated
MAPK signaling pathway
Focal adhesion
Cell adhesion molecules
Leukocyte transendothelial migration
No. of genes
F
RUNX1 lost - down-regulated
Cell cycle
RNA transport
Ribosome biogenesis in eukaryotes
p53 signaling pathway
No. of genes
G
Log10 normalised counts
CD34+ PBSCs
Primary t(8;21)
FLT1
FLT4
KDR
VEGFA
t(8;21) PDX siRUNX1-ETO
t(8;21) PDX siMM
H
Ranked by 10Dox/0Dox fold difference
1996
23189
5534
ESC-derived RUNX1c+ cells
0Dox ATAC
5Dox ATAC
10Dox ATAC
5Dox ETO ChIP
RUNX1-ETO+ cord blood cells
ATAC
ETO ChIP
Primary t(8;21) cells
PatientB ETO ChIP

Supplementary Figure 3 (related to Figure 3)

RUNX1-ETO interferes with the RUNX1-driven gene regulatory network

A., B. UCSC browser screenshots showing RUNX1 and RUNX1-ETO binding at the *Vav1* (A) locus and the *VEGFA* locus (B) in the presence or absence of 5 ng/ml Dox.

C.- F. The same analysis as in Fig. 3 C.- F. but for cells induced with 10 ng/ml Dox.

G: Bar diagram depicting the fold-change in expression of endothelial genes in CD34+ cells from t(8;21) patients as compared to PBSCs [2] and in t(8;21) PDX cells after knock-down of RUNX1-ETO (siRE) compared to the control (siMM) [3])

H. Comparison of RUNX1-ETO ChIP-Seq data from different sources. Left panel: ESC-derived data. 0 Dox ATACSeq peak counts (red) ranked by the difference between 0 Dox and 10 ng/ml Dox with 1996 peaks gained and 5534 peaks lost after induction of RUNX1-ETO. RUNX1-ETO ChIP peaks after induction with 5 ng/ml Dox are plotted alongside the same ranked genomic coordinates (blue). Middle panel: ATACseq (red) and ChIPseq (blue) data from RUNX1-ETO transduced cord blood cells [4] are plotted along-side the ESC ATAC data. Right panel: ChIP data (blue) from a t(8;21) patient [5] plotted along-side. Patient B ChIP has:3568 peaks, of which 2618 (73%) are shared with the cord blood ETO ChIP and 1452 (41%) are shared with the 5 ng/ml Dox RUNX1c+ ETO ChIP. 1435 (40%) are in all 3.

Figure S4 revised

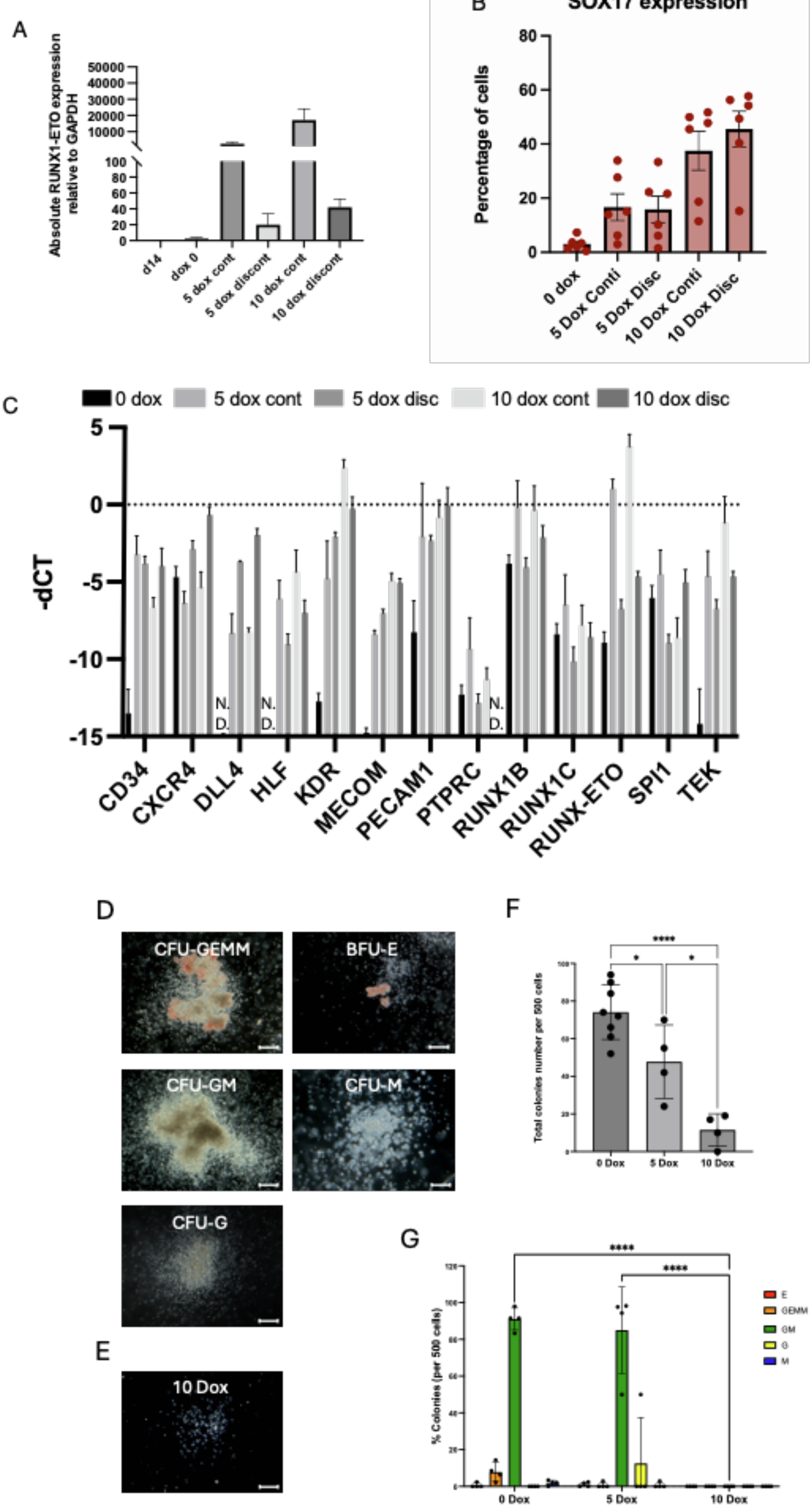

Supplementary Figure 4 (related to Figure 4)

Induction of RUNX1-ETO interferes with hematopoietic colony formation, proliferation and differentiation

A: Real-Time gene expression analysis determining the absolute value of RUNX1-ETO expression before induction, after induction with 5 or 10 ng/ml of Dox and after Dox withdrawal in Matrigel cultures. Error bars represent S.E.M of n=3

B. Percentage of cells expressing the SOX17 reporter after induction and withdrawal of 5 ng/ml and 10 ng/ml Dox. Error bars represent S.E.M. n =6.

C: Real-time PCR gene expression analysis of the indicated genes from cells at the end of Matrigel culture in the presence or absence of Dox. Error bars represent S.E.M. n =3 or n=6.

D: Representative photos of blood colonies CFU-GEMM (granulocyte, erythrocyte, monocyte, megakaryocyte), BFU-E (erythrocyte), CFU-GM (granulocyte /macrophage), CFU-G (granulocyte), and CFU-M (macrophage) as indicated. The bar represents 200 micrometres.

E: Representative photo of a colony of undifferentiated cells treated with 10 ng/mL Dox for 15 days. The bar represents 200 micrometres.

F: Total number of colonies per 500 cells plated without or with 5 and 10 ng/ml Dox.

G: **C**olony types quantified as CFU-GEMM (granulocyte, erythrocyte, monocyte, megakaryocyte), CFU-GM (granulocyte/macrophage), CFU-G (granulocyte), and CFU-M (macrophage), expressed as a percentage of total colonies found in (**C**). Statistical significance was assessed using the Ordinary one-way ANOVA test followed by Tukey's multiple comparisons test (**C**) or using the 2way ANOVA test followed by Tukey's multiple comparisons test (**D**). *$p < 0.05$; ****$p<0.0001$.

Figure S5 revised

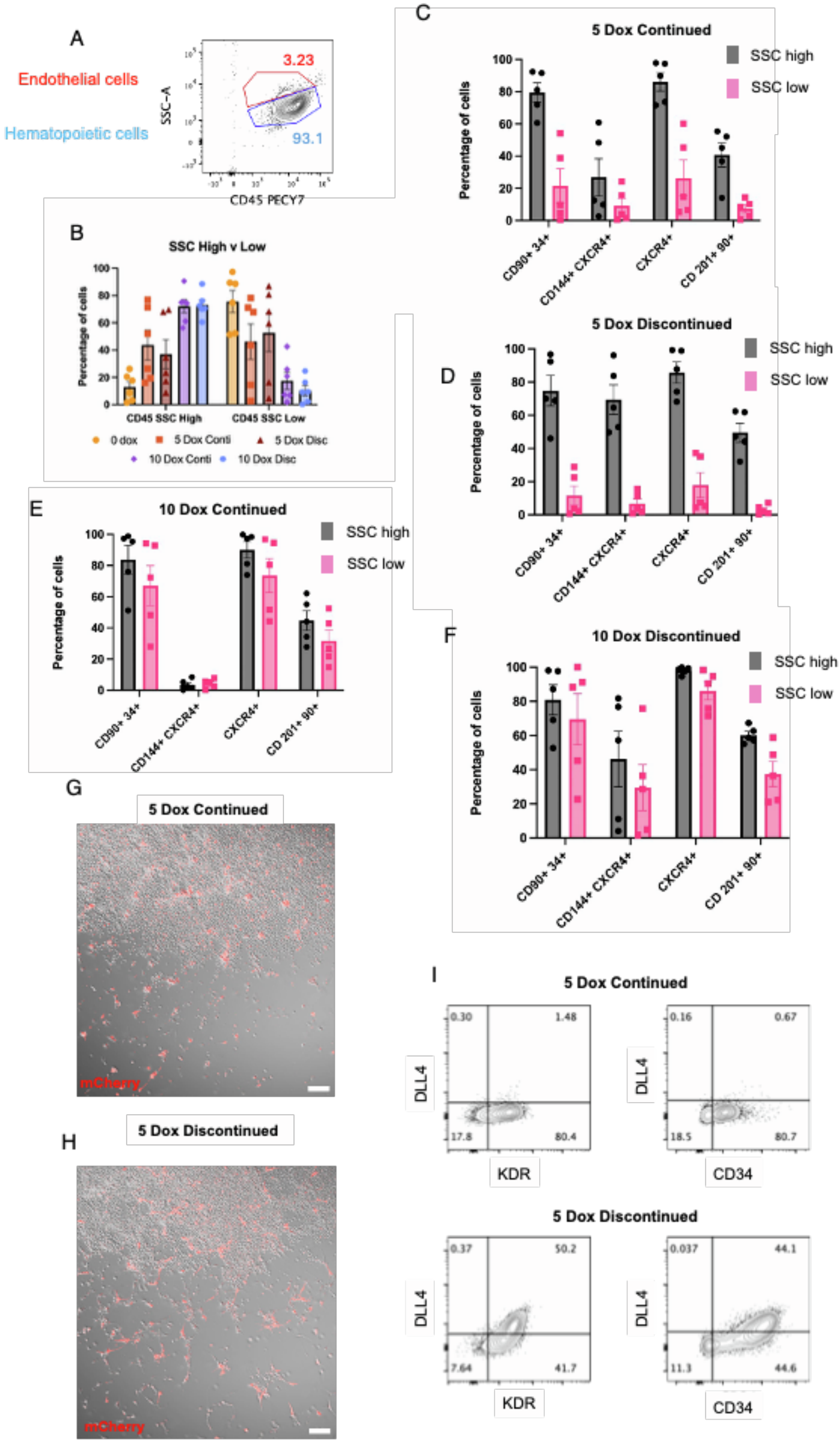

Supplementary Figure 5 (related to Figure 5)

Induction of RUNX1-ETO generates endothelial cells in a dose-dependent and irreversible fashion

A: Outline of the FACS sorting strategy for CD90+ endothelial cells reduced CD45 expression and with increased SSC, in this case from a population with mainly hematopoietic cells. Cells were harvested 48hours after being plated in the Matrigel assay.

B. Analysis of CD45+ cells according to their size (SSC) previously induced with 5 or 10 ng/ml Dox with or without Dox in the Matrigel culture as compared to the 0 Dox control. Error bars represent S.E.M. of n=6

C.- F. Quantitative analysis of Flow cytometry analyses shown in Fig. 3 G. – K. Analysis of hematopoietic and endothelial cells expressing the indicated surface markers induced with 5 ng/ml Dox with continuation of Dox in the Matrigel culture (C), analysis of cells induced with 5 ng/ml Dox without Dox in the Matrigel culture (D), analysis of cells induced with 10 ng/ml Dox with continuation of Dox in the Matrigel culture (E), analysis of cells induced with 10ng/ml Dox without Dox in the Matrigel culture (F). Error bars represent s.e.m. of n=5

G, H: Confocal images of representative Matrigel culture continuously cultured in 10 ng/ml Dox **(G, cont)** or where Dox was withdrawn (**H, Disc)** with the red colour coming from the SOX17 reporter expressing mCherry. Scale bar represents 100 micrometres.

I: Intracellular FACS staining of the NOTCH ligand DLL4 and surface expression of the VEGF receptor FLK1 in isolated adherent endothelial-like cells from Matrigel cultures of cell induced with 5 ng/ml Dox after 4 days of culture with (Dox continued, upper panels) and without (Dox discontinued, lower panels) Dox.

Figure S6 revised

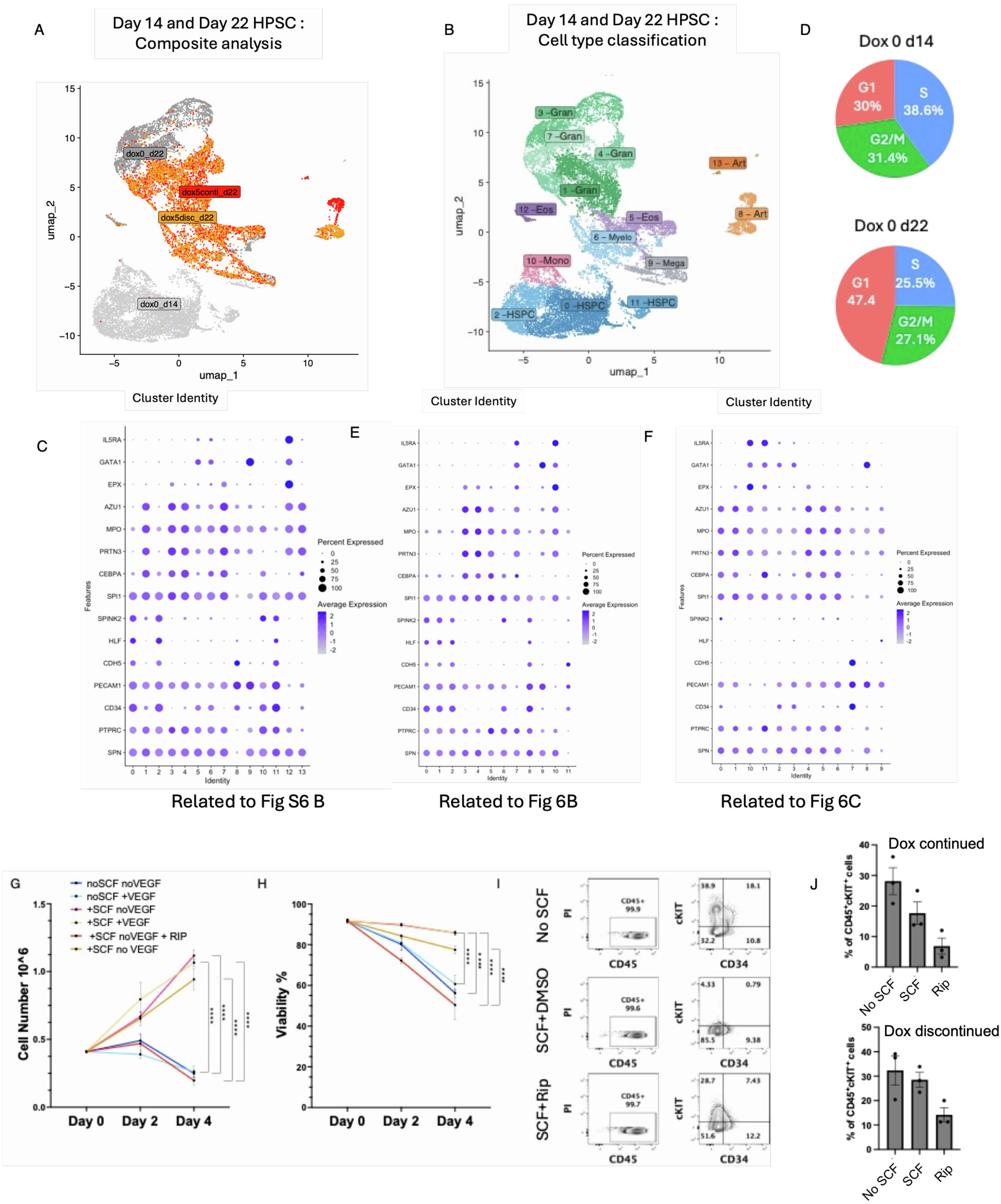

Supplementary Figure 6 (related to Figure 6)

Endothelial culture supports the proliferation of hematopoietic cells

A. UMAP representation of scRNA-Seq data depicting Seurat clusters representing day 14 HPSCs (starting material), cells from the Matrigel cultures at Day 22 with continuing induction with 5 or 10 ng/ml or cells where Dox was withdrawn.

B. UMAP representation of the same cell populations as in A., annotating cell types to 13 clusters. Myelo: Myeloid progenitor cells, Art: Arterial endothelium, Gran: maturing Granulocytic cells, Eos: Eosinophils, Mega: Megakaryocytes, Mono: Monocytes.

C. Dot plot showing expression levels of differentially expressed genes in cell annotated to Seurat clusters depicted in Figure S6B representing arterial, hemogenic endothelium and hematopoietic genes.

D. Cell cycle status of uninduced Day 14 and Day 22 populations.

E. Dot plot of expression levels of differentially expressed genes in cells allocated to the Seurat clusters depicted in Figure 6B representing stem cell and hematopoietic lineage genes.

F. Dot plot of expression levels of differentially expressed genes in cells allocated to the Seurat clusters depicted in Figure 6C representing stem cell and hematopoietic lineage genes.

G,H. Day15 HSPCs were induced with 5 ng/ml Dox for 6 days, placed into Matrigel medium for 4 days with or without VEGF, SCF or both, or with SCF plus 64 µM Ripretinib, followed by measuring cell numbers (Fig S6 G) and viability (Fig S6H). Viability was measured by Propidium Iodide (PI) staining (see Methods). Data represent mean ± SEM from n = 3 independent experiments. Statistical significance was assessed using the 2way ANOVA test followed by an uncorrected Fisher's LSD, ****$p<0.0001$.

I. FACS analyses examining KIT, CD34 and CD45 expression on cells from G grown in Matrigel medium without SCF, with SCF plus DMSO and SCF+ Ripretinib as indicated. The outer left panel shows gating on life cells.

J. Bar graph depicting the combined results of three independent Matrigel culture experiments measuring KIT+/CD45+ cells under the indicated conditions as shown in Figure 7 B,C. Error bars represent s.e.m n=3

Figure S7

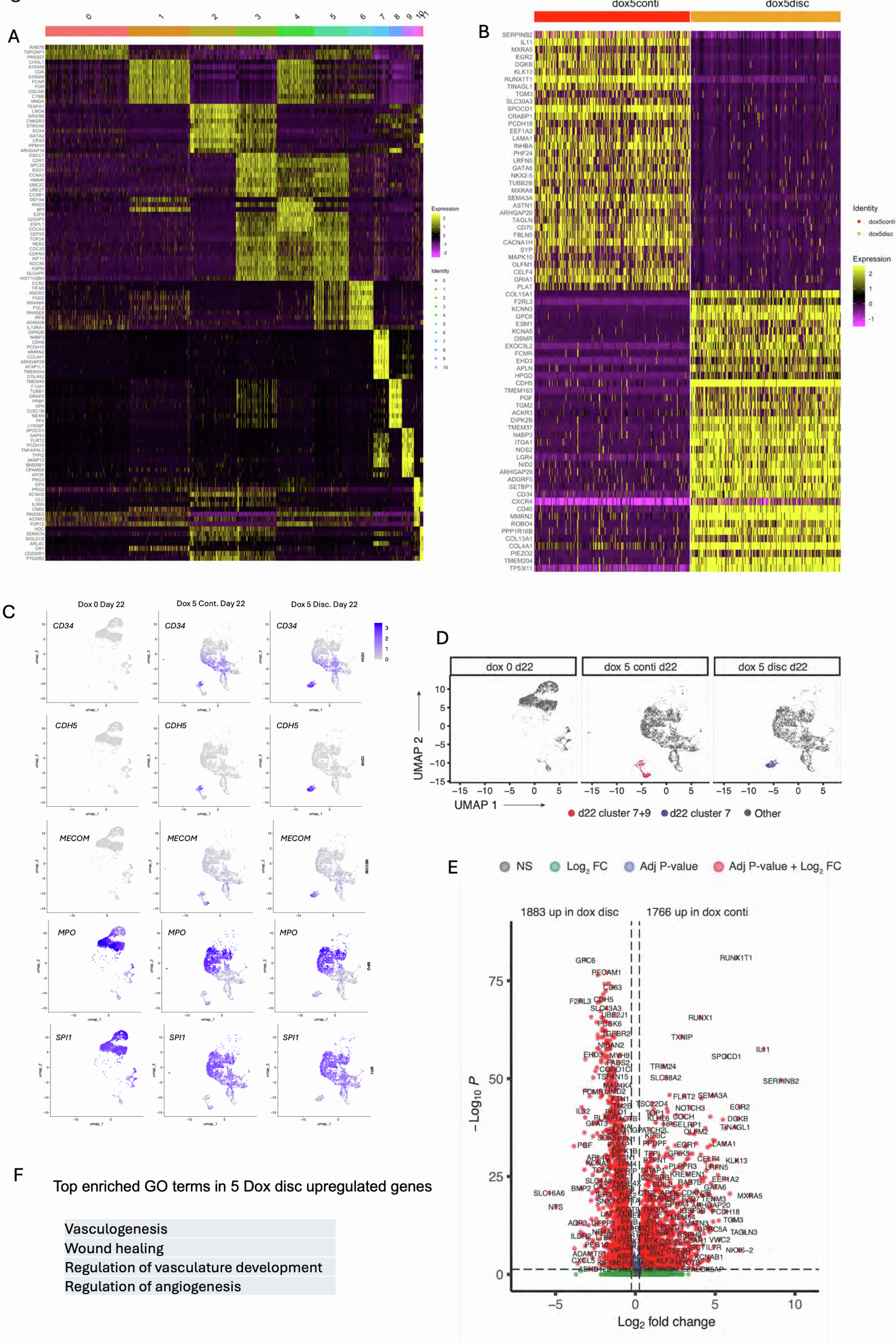
A
B
dox5conti
dox5disc
Expression
Identity
C
Dox 0 Day 22
Dox 5 Cont. Day 22
Dox 5 Disc. Day 22
CD34
CDH5
MECOM
MPO
SPI1
D
dox 0 d22
dox 5 conti d22
dox 5 disc d22
UMAP 2
UMAP 1
d22 cluster 7+9
d22 cluster 7
Other
E
NS
Log2 FC
Adj P-value
Adj P-value + Log2 FC
1883 up in dox disc
1766 up in dox conti
− Log10 P
Log2 fold change
F
Top enriched GO terms in 5 Dox disc upregulated genes
Vasculogenesis
Wound healing
Regulation of vasculature development
Regulation of angiogenesis

Supplementary Figure 7 (related to Figure 6)

Dox withdrawal releases the RUNX1-ETO mediated block in endothelial differentiation

A. Heatmap of cluster representative genes used to determine sample identity based on human embryo data as in [1]. The purple colour represents low, and the yellow colour represents high level expression compared to all other samples.

B. Heatmap of cluster representative genes discriminating between d22 cells that received continued induction with 5ng/ml Dox (dox5 conti) or where Dox was withdrawn (dox5 disc).

C. Feature plots from the same cell populations as in (Fig.1 E) separated by their induction status (Dox 0: No induction), continued induction with 5 ng/ml Dox and after Dox withdrawal (Discont.) showing the expression levels of the indicated genes in the different cell populations.

D. UMAP of scRNA-seq data from day 22 split by doxycycline condition. Arterial populations from dox 5 continuous and 5 dox discontinued populations highlighted red and blue respectively.

E. Volcano plot showing differentially expressed genes between the Dox 5 continuous and Dox 5 discontinued arterial populations. Genes were considered differentially expressed if they had an absolute log fold-change ≥ 0.25 and an adjusted p-value < 0.05.

F: List of top enriched Gene Ontology terms in genes upregulated after withdrawal of 5 ng/ml Dox.

References

1. Ng, E.S., Sarila, G., Li, J.Y., Edirisinghe, H.S., Saxena, R., Sun, S., Bruveris, F.F., Labonne, T., Sleebs, N., Maytum, A., et al. (2024). Long-term engrafting multilineage hematopoietic cells differentiated from human induced pluripotent stem cells. Nat Biotechnol. 10.1038/s41587-024-02360-7.
2. Assi, S.A., Imperato, M.R., Coleman, D.J.L., Pickin, A., Potluri, S., Ptasinska, A., Chin, P.S., Blair, H., Cauchy, P., James, S.R., et al. (2019). Subtype-specific regulatory network rewiring in acute myeloid leukemia. Nat Genet *51*, 151-162. 10.1038/s41588-018-0270-1.
3. Derevyanko, P.K., Swart, L.E., Mata Casimiro, L.D., van Oort, A., du Plessis, M., van den Brink, L., Ashtiani, M., Zwaan, C.M., Krippner-Heidenreich, A., Bonifer, C., et al. (2025). Fusion gene depletion eliminates stemness and induces bidirectional differentiation of acute myeloid leukemia. Blood *146*, 2963-2978. 10.1182/blood.2025028988.
4. Chin, P.S., Assi, S.A., Ptasinska, A., Imperato, M.R., Cockerill, P.N., and Bonifer, C. (2020). RUNX1/ETO and mutant KIT both contribute to programming the transcriptional and chromatin landscape in t(8;21) acute myeloid leukemia. Exp Hematol *92*, 62-74. 10.1016/j.exphem.2020.10.005.
5. Ptasinska, A., Assi, S.A., Mannari, D., James, S.R., Williamson, D., Dunne, J., Hoogenkamp, M., Wu, M., Care, M., McNeill, H., et al. (2012). Depletion of RUNX1/ETO in t(8;21) AML cells leads to genome-wide changes in chromatin structure and transcription factor binding. Leukemia *26*, 1829-1841. 10.1038/leu.2012.49.
